# An efficient, not-only-linear correlation coefficient based on clustering

**DOI:** 10.1016/j.cels.2024.08.005

**Published:** 2024-09-06

**Authors:** Milton Pividori, Marylyn D. Ritchie, Diego H. Milone, Casey S. Greene

**Affiliations:** 1 Department of Biomedical Informatics, University of Colorado School of Medicine, Aurora, CO 80045, USA; 2 Department of Genetics, Perelman School of Medicine, University of Pennsylvania, Philadelphia, PA 19104, USA; 3 Research Institute for Signals, Systems and Computational Intelligence (sinc(i)), Universidad Nacional del Litoral, Consejo Nacional de Investigaciones Científicas y Técnicas (CONICET), Santa Fe CP3000, Argentina; 4 Center for Health AI, University of Colorado School of Medicine, Aurora, CO 80045, USA; 5 Lead contact

## Abstract

Identifying meaningful patterns in data is crucial for understanding complex biological processes, particularly in transcriptomics, where genes with correlated expression often share functions or contribute to disease mechanisms. Traditional correlation coefficients, which primarily capture linear relationships, may overlook important nonlinear patterns. We introduce the clustermatch correlation coefficient (CCC), a not-only-linear coefficient that utilizes clustering to efficiently detect both linear and nonlinear associations. CCC outperforms standard methods by revealing biologically meaningful patterns that linear-only coefficients miss and is faster than state-of-the-art coefficients such as the maximal information coefficient. When applied to human gene expression data from genotype-tissue expression (GTEx), CCC identified robust linear relationships and nonlinear patterns, such as sex-specific differences, that are undetectable by standard methods. Highly ranked gene pairs were enriched for interactions in integrated networks built from protein-protein interactions, transcription factor regulation, and chemical and genetic perturbations, suggesting that CCC can detect functional relationships missed by linear-only approaches. CCC is a highly efficient, next-generation, not-only-linear correlation coefficient for genome-scale data. A record of this paper’s transparent peer review process is included in the supplemental information.

## In brief

Correlation coefficients are widely used to identify patterns. We introduce CCC, an efficient, easy-to-use coefficient based on clustering that reveals biologically meaningful linear and nonlinear patterns. When applied to human gene expression data, CCC identifies nonlinear patterns explained by sex differences that are not captured by standard and linear-only coefficients.

## INTRODUCTION

New technologies have vastly improved data collection, generating a deluge of information across different disciplines. This large amount of data provides new opportunities to address unanswered scientific questions, provided we have efficient tools capable of identifying multiple types of underlying patterns. Correlation analysis is an essential statistical technique for discovering relationships between variables.^1^ Correlation coefficients are often used in exploratory data mining techniques, such as clustering or community detection algorithms, to compute a similarity value between a pair of objects of interest, such as genes^2^ or disease-relevant lifestyle factors.^3^ These coefficients are also used in supervised tasks, for example, for feature selection to improve prediction accuracy.^4,5^ The Pearson correlation coefficient is ubiquitously deployed across application domains and diverse scientific areas. Thus, even minor and significant improvements in these techniques could have enormous consequences in industry and research.

In transcriptomics, many analyses start with estimating the correlation between genes. More sophisticated approaches built on correlation analysis can suggest gene function,^6^ aid in discovering common and cell lineage-specific regulatory networks,^7^ and capture important interactions in a living organism that can uncover molecular mechanisms in other species.^8,9^ The analysis of large RNA-sequencing (RNA-seq) datasets^10,11^ can also reveal complex transcriptional mechanisms underlying human diseases.^2,12-15^ Since the introduction of the omnigenic model of complex traits,^16,17^ gene-gene relationships are playing an increasingly important role in genetic studies of human diseases,^15,18-20^ even in specific fields such as polygenic risk scores.^21^ In this context, recent approaches combine disease-associated genes from genome-wide association studies (GWASs) with gene co-expression networks to prioritize “core” genes directly affecting diseases.^19,20,22^ These core genes are not captured by standard statistical methods but are believed to be part of highly interconnected, disease-relevant regulatory networks. Therefore, advanced correlation coefficients could immediately find wide applications across many areas of biology, including the prioritization of candidate drug targets in the precision medicine field.

The Pearson and Spearman correlation coefficients are widely used because they reveal intuitive relationships and can be computed quickly. However, they are designed to capture linear or monotonic patterns (referred to as linear-only) and may miss complex yet critical relationships. Novel coefficients have been proposed as metrics that capture nonlinear patterns, such as the maximal information coefficient (MIC)^23^ and the distance correlation (DC).^24^ MIC, in particular, is one of the most commonly used statistics to capture more complex relationships, with successful applications across several domains.^4,25,26^ However, the computational complexity makes them impractical for even moderately sized datasets.^25,27^ Recent implementations of MIC, for example, take several seconds to compute on a single variable pair across a few thousand objects or conditions.^25^ We previously developed a clustering method for highly diverse datasets that outperformed approaches based on Pearson, Spearman, DC, and MIC in detecting clusters of simulated linear and nonlinear relationships with varying noise levels.^28^

Here we introduce the clustermatch correlation coefficient (CCC), an efficient, not-only-linear coefficient that works across quantitative and qualitative variables. CCC has a single parameter that limits the maximum complexity of relationships found (from linear to more general patterns) and computation time. CCC provides a high level of flexibility to detect specific types of patterns that are more important for the user while providing safe defaults to capture general relationships. We also provide an efficient CCC implementation that is highly parallelizable, allowing us to speed up computation across variable pairs with millions of objects or conditions. To assess its performance, we applied our method to gene expression data from the genotype-tissue expression (GTEx) v8 project across different tissues.^10^ CCC captured both strong linear relationships and novel nonlinear patterns, which were entirely missed by standard coefficients. For example, some of these nonlinear patterns were associated with sex differences in gene expression, suggesting that CCC can capture strong relationships present only in a subset of samples. We also found that the CCC behaves similarly to MIC in several cases, although it is much faster to compute. Gene pairs detected in expression data by CCC had higher interaction probabilities in tissue-specific gene networks from the genome-wide integrated analysis of gene networks in tissues (GIANT).^7^ Furthermore, its ability to efficiently handle diverse data types (including numerical and categorical features) reduces preprocessing steps and makes it appealing to analyze large and heterogeneous repositories.

## RESULTS

### Overview of CCC: A not-only-linear correlation coefficient

The CCC provides a similarity measure between any pair of variables, either with numerical or categorical values. The method assumes that if there is a relationship between two variables/features describing n data points/objects, then the clusterings of those objects using each variable should match. In the case of numerical values, CCC uses quantiles to efficiently separate data points into different clusters (e.g., the median separates numerical data into two clusters). For categorical values, CCC uses the categories themselves to separate data points into different clusters (e.g., if feature color has three values, red, green, and blue, then data will be clustered into three clusters defined by those colors). Once all clusterings are generated according to each variable, we define the CCC as the maximum adjusted Rand index (ARI)^29^ between them, ranging between 0 and 1. Details of the CCC algorithm can be found in Box 1.

**Figure 1.**
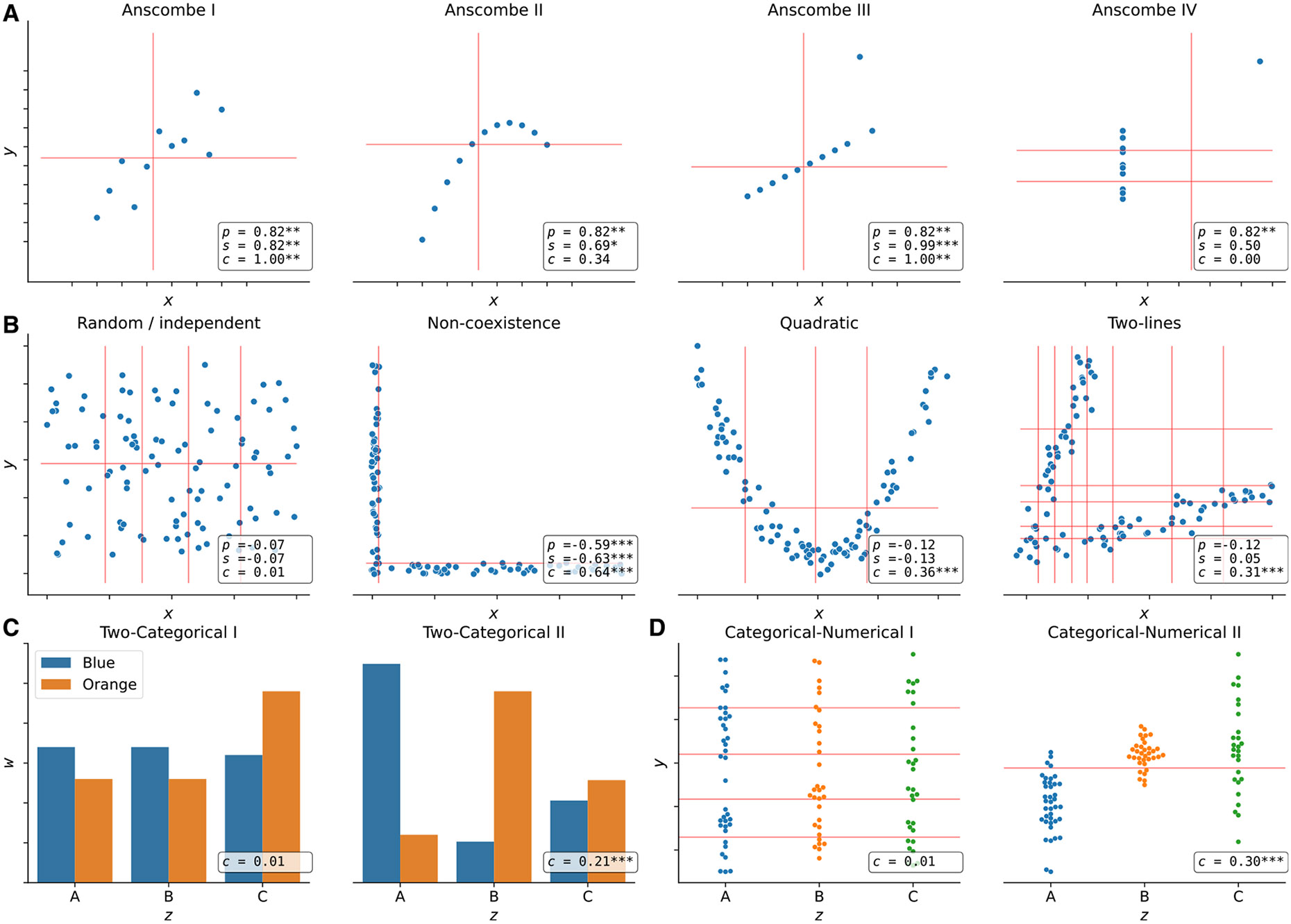
Different patterns across data types Each panel contains a set of simulated datasets described by two generic variables. (A) The Anscombe’s quartet with four different datasets (from Anscombe I to IV) with numerical variables x and y and 11 data points. (B) Four datasets with 100 data points. (C) Two datasets with categorical variables w (with values orange and blue) and z (with values A, B, and C) and 100 data points. (D) Two datasets with categorical and numerical variables and 100 data points. Each panel shows the correlation value using Pearson (p) and Spearman (s) for numerical variables only and CCC (c) for both numerical and categorical, and their statistical significance is indicated with asterisks: *p* < 0:05 (*), *p* < 0:01 (**), and *p* < 0:001 (***). For CCC, we computed the p value using 10,000 permutations. Vertical and horizontal red lines show how CCC clustered data points using x and y, respectively. For categorical variables, CCC uses the categories to cluster data points.

We examined how the Pearson (p), Spearman (s), and CCC (c) correlation coefficients behaved on different simulated data patterns. Figure 1 shows different types of relationships between two variables of different data types, where x and y are numerical and w and z are categorical. For each variable pair, we show the coefficient values and their statistical significance, where asterisks indicate different p values (p). The red lines show how CCC clustered numerical data points using x (vertical lines) and y (horizontal lines).

In Figure 1A, we examine the classic Anscombe’s quartet,^30^ which comprises four synthetic datasets with different patterns but the same data statistics (mean, standard deviation, and Pearson’s correlation). This kind of simulated data, recently revisited with the “Datasaurus,”^31,32^ is used as a reminder of the importance of going beyond simple statistics, where either undesirable patterns (such as outliers) or desirable ones (such as biologically meaningful nonlinear relationships) can be masked by summary statistics alone. Anscombe I contains a noisy but clear linear pattern, similar to Anscombe III, where the linearity is perfect besides one outlier. In these two examples, CCC separates data points using two clusters (one red line for each variable x and y), yielding a statistically significant value of 1:0 (the maximum for CCC) and thus indicating a strong relationship. Anscombe II seems to follow a partially quadratic relationship, interpreted as linear by Pearson and Spearman. By contrast, for this potentially undersampled quadratic pattern, CCC yields a lower and not statistically significant value of 0:34, reflecting a more complex relationship than a linear pattern. Anscombe IV shows a vertical line of data points where x values are almost constant except for one outlier. This outlier does not influence CCC (which correctly identifies no relationship) as it does for Pearson or Spearman, although only Pearson yields a statistically significant result. Thus, c=0.00 (the minimum value) correctly indicates no association for this variable pair because, besides the outlier, for a single value of x, there are ten different values for y. This pair of variables does not fit the CCC assumption: the two clusters formed with x (approximately separated by x=13) do not match the three clusters formed with y. The Pearson’s correlation coefficient is the same across all of Anscombe’s examples (*p* = 0:82), whereas Spearman’s is 0:50 or greater.

We also simulated additional types of numerical relationships (Figure 1B), including some previously described from gene expression data.^23,33,34^ For the random/independent pair of variables, all coefficients correctly agree with a value close to zero and *p* > 0:05. The non-coexistence pattern, captured by all coefficients, represents a case where one gene (x) might be expressed while the other one (y) is inhibited, highlighting a potentially strong biological relationship (such as a microRNA negatively regulating another gene). For the other two examples (quadratic and two-lines), only CCC is able to yield a high and statistically significant correlation value, whereas Pearson and Spearman fail to capture these nonlinear patterns. These relationships also show how CCC uses different degrees of complexity to capture the relationships. For the quadratic pattern, for example, CCC separates x into more clusters (four in this case) to reach the maximum ARI. The two-lines example shows two embedded linear relationships with different slopes, which neither Pearson nor Spearman detects (*p* = − 0:12 and s=0.05, respectively, both with *p* > 0:05). Here, CCC increases the complexity of the model by using eight clusters for x and six for y, resulting in c=0.31 (*p* < 0:001).

Furthermore, we also simulated categorical variables, which only CCC can handle. Figure 1C shows two patterns between variables w (with categories orange and blue) and z (with categories A, B, and C). The first case (two-categorical I) represents a random/independent pattern where categorical values in one variable are approximately uniformly distributed across the categorical values of the other variable. Here, as expected, CCC yields a very low and non-significant value. In the second case (two-categorical II), the category blue of w is overrepresented in data points with z equal to A and, less strongly, the category orange of w is overrepresented in data points with z equal to B. In this case, since CCC clusters data points using the categorical values, it detects that clusters of data points with w = blue match clusters with z=A, yielding a statistically significant c=0.21. Figure 1D mixes a categorical variable (z) with a numerical one (y). The first case (categorical-numerical I) represents a random/independent pattern where numerical values in y are approximately uniformly distributed across the categorical values in z. Similarly, as in the other random/independent cases, CCC yields a very low and non-significant value since the clusters formed by y do not match the clusters (given by the categorical values) formed by z. Conversely, in the second case (categorical-numerical II), clusters of data points with similar values in y tend to have also similar categorical values in z. In this example, for data points with z=A, we assigned $y∼N(0,0.5^{2})$, whereas for z=B and C, we assigned $y∼N(1,0.25^{2})$ and $y∼N(1,0.75^{2})$, respectively. Here, CCC uses y values to group data points into two clusters, and these clusters match the clusters obtained from z, yielding a statistically significant c=0.30.

### The CCC reveals linear and nonlinear patterns in human transcriptomic data

We next examined the characteristics of these correlation coefficients in gene expression data from GTEx v8 across different tissues. For our initial analyses, we selected the top 5,000 genes with the largest variance on whole blood and then computed the correlation matrix between genes using Pearson, Spearman, and CCC (see STAR Methods). Although we always considered the statistical significance of the coefficients, we focused on the strength of the association (i.e., the coefficient value) for our analyses.

**Figure 2.**
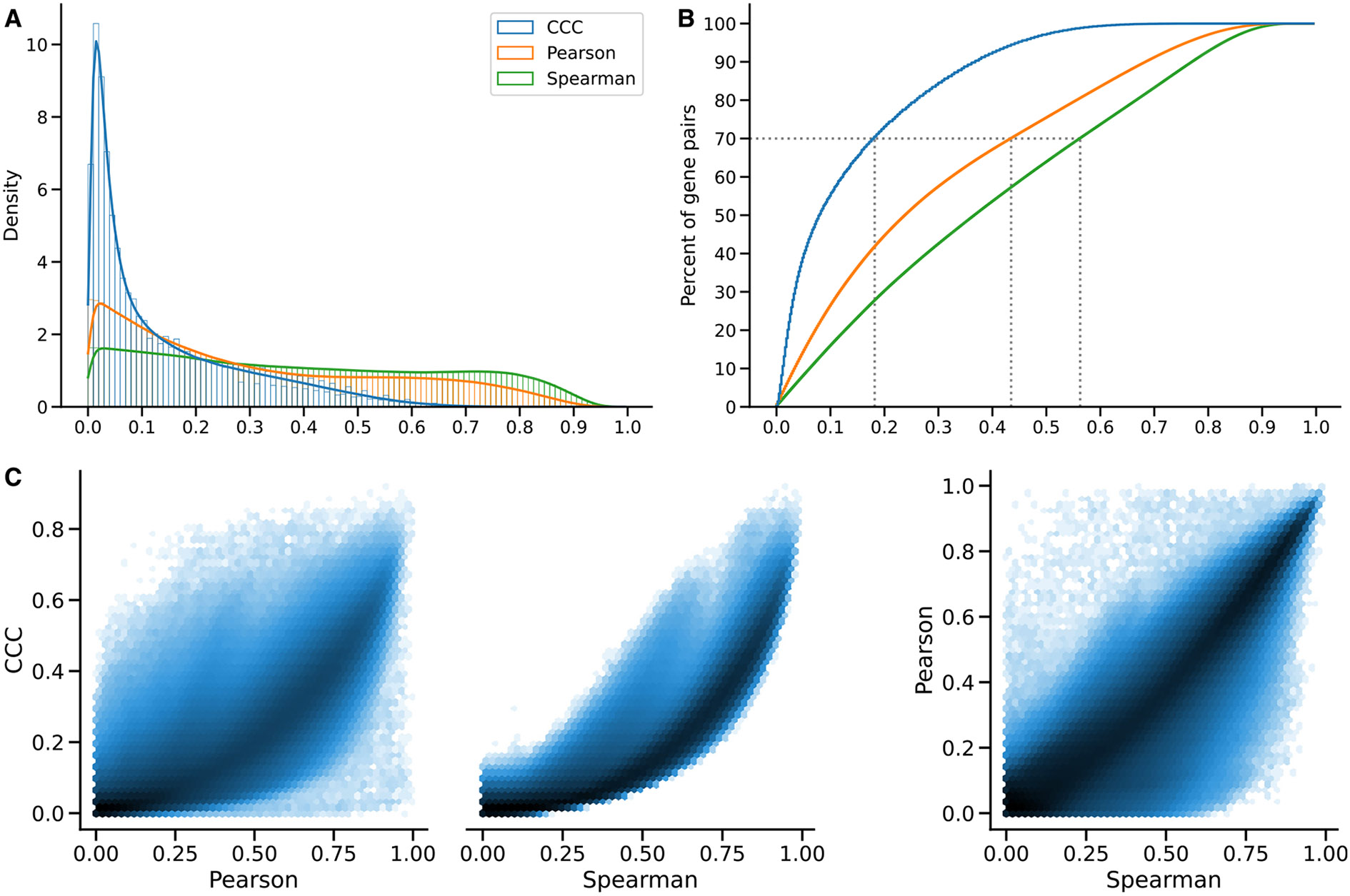
Distribution of coefficient values on gene expression (GTEx v8, whole blood) (A) Histogram of coefficient values. (B) Corresponding cumulative histogram. The dotted line maps the coefficient value that accumulates 70% of gene pairs. (C) 2D histogram plot with hexagonal bins between all coefficients, where a logarithmic scale was used to color each hexagon.

We examined the distribution of each coefficient’s absolute values in GTEx (Figure 2). CCC (mean = 0.14, median = 0.08, SD = 0.15) has a much more skewed distribution than Pearson (mean = 0.31, median = 0.24, SD = 0.24) and Spearman (mean = 0.39, median = 0.37, SD = 0.26). The coefficients reach a cumulative set containing 70% of gene pairs at different values (Figure 2B), c=0.18, *p* = 0:44, and s=0.56, suggesting that for this type of data, the coefficients are not directly comparable by magnitude, so we used ranks for further comparisons. In GTEx v8, CCC values were closer to Spearman than either was to Pearson (Figure 2C). As shown later, we also compared with the MIC, another advanced, not-only-linear correlation coefficient that has been successfully used in various application domains.^4,25,26^ We found that CCC behaved very similarly to MIC, although CCC was up to two orders of magnitude faster to run. These results suggest that our findings for CCC generalize to MIC; therefore, in the subsequent analyses, we focus on CCC and linear-only coefficients.

**Figure 3.**
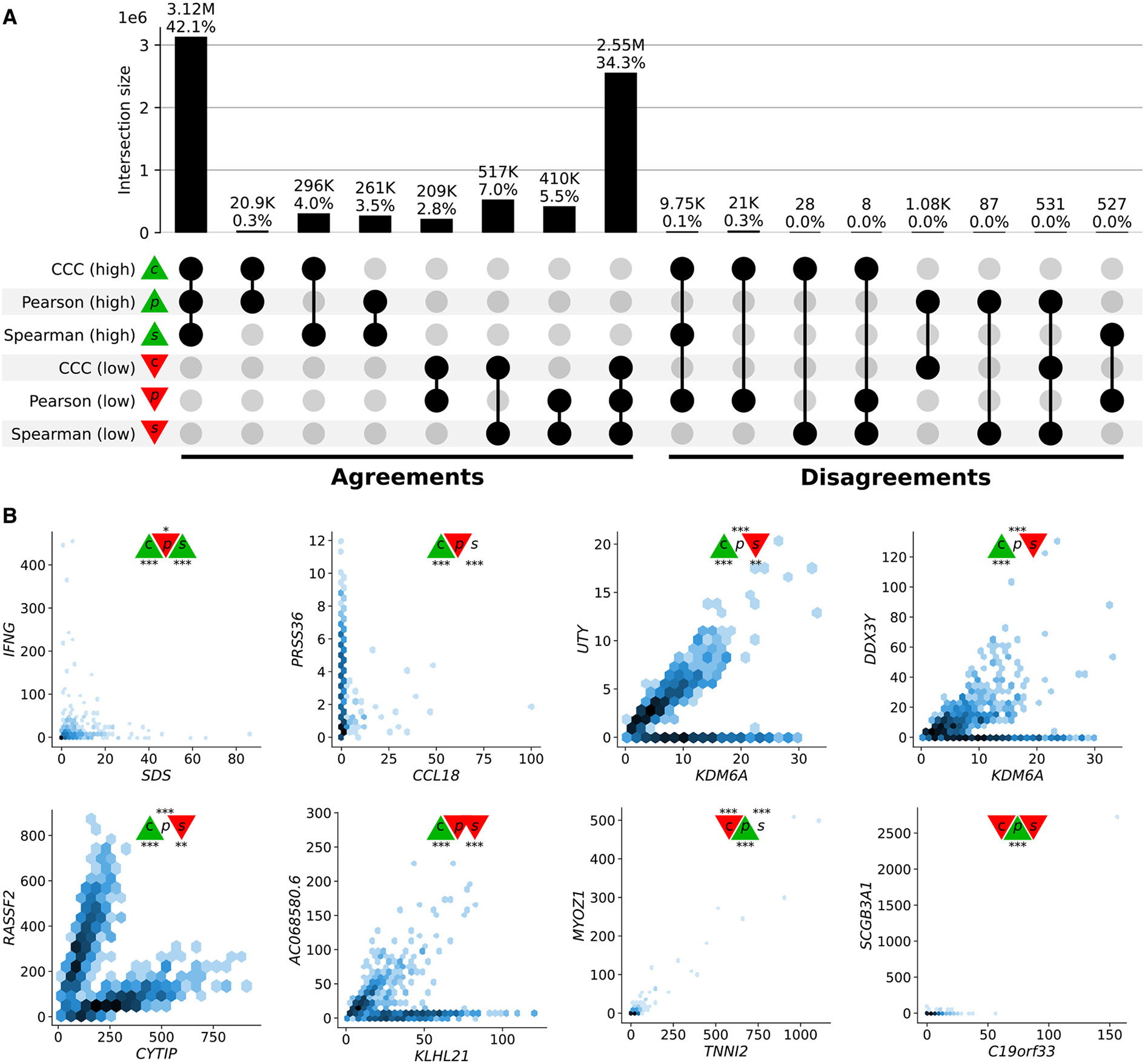
Intersection of gene pairs with high and low correlation coefficient values (GTEx v8, whole blood) (A) UpSet plot with six categories (rows) grouping the 30% of the highest (green triangle) and lowest (red triangle) values for each coefficient. Columns show different intersections of categories grouped by agreements and disagreements. (B) Hexagonal binning plots with examples of gene pairs where CCC (c) disagrees with Pearson (p) and Spearman (s). For each method, colors in the triangles indicate if the gene pair is among the top (green) or bottom (red) 30% of coefficient values. No triangle means that the correlation value for the gene pair is between the 30th and 70th percentiles (neither low nor high). The statistical significance is indicated with asterisks using the false discovery rate (FDR) adjusted p values, calculated using the Benjamini and Hochberg method^36^: *FDR* < 0:05 (*), *FDR* < 0:01 (**), and *FDR* < 0:001 (***). A logarithmic scale was used to color each hexagon.

A closer inspection of gene pairs that were either prioritized or disregarded by these coefficients revealed that they captured different patterns. We analyzed the agreements and disagreements by obtaining, for each coefficient, the top 30% of gene pairs with the largest correlation values (“high” set) and the bottom 30% (“low” set), resulting in six potentially overlapping categories (see “All pairwise gene correlations in GTEx whole blood” and “Coefficients percentiles in GTEx whole blood” in the key resources table). For most cases (76.4%), an UpSet analysis^35^ (Figure 3A) showed that the three coefficients agreed on whether there is a strong correlation (42.1%) or there is no relationship (34.3%). Since Pearson and Spearman are linear-only, and CCC can also capture these patterns, we expect that these concordant gene pairs represent clear linear patterns. CCC and Spearman agree more on either highly or poorly correlated pairs (4.0% in high and 7.0% in low) than any of these with Pearson (all between 0.3%–3.5% for high and 2.8%–5.5% for low). In summary, CCC agrees with either Pearson or Spearman in 90.5% of gene pairs by assigning a high or a low correlation value.

While there was broad agreement, more than 20,000 gene pairs with a high CCC value were not highly ranked by the other coefficients (“disagreements” group on the right of Figure 3A). There were also gene pairs with a high Pearson value and either low CCC (1,075), low Spearman (87), or both low CCC and low Spearman values (531). No gene pairs were found to have a high Spearman value and a low CCC. Considering the correlation values and their statistical significance, we analyzed gene pairs among the top ten of each intersection in the disagreements group (Figure 3A, right) where CCC disagrees with Pearson, Spearman, or both.

**Figure 4.**
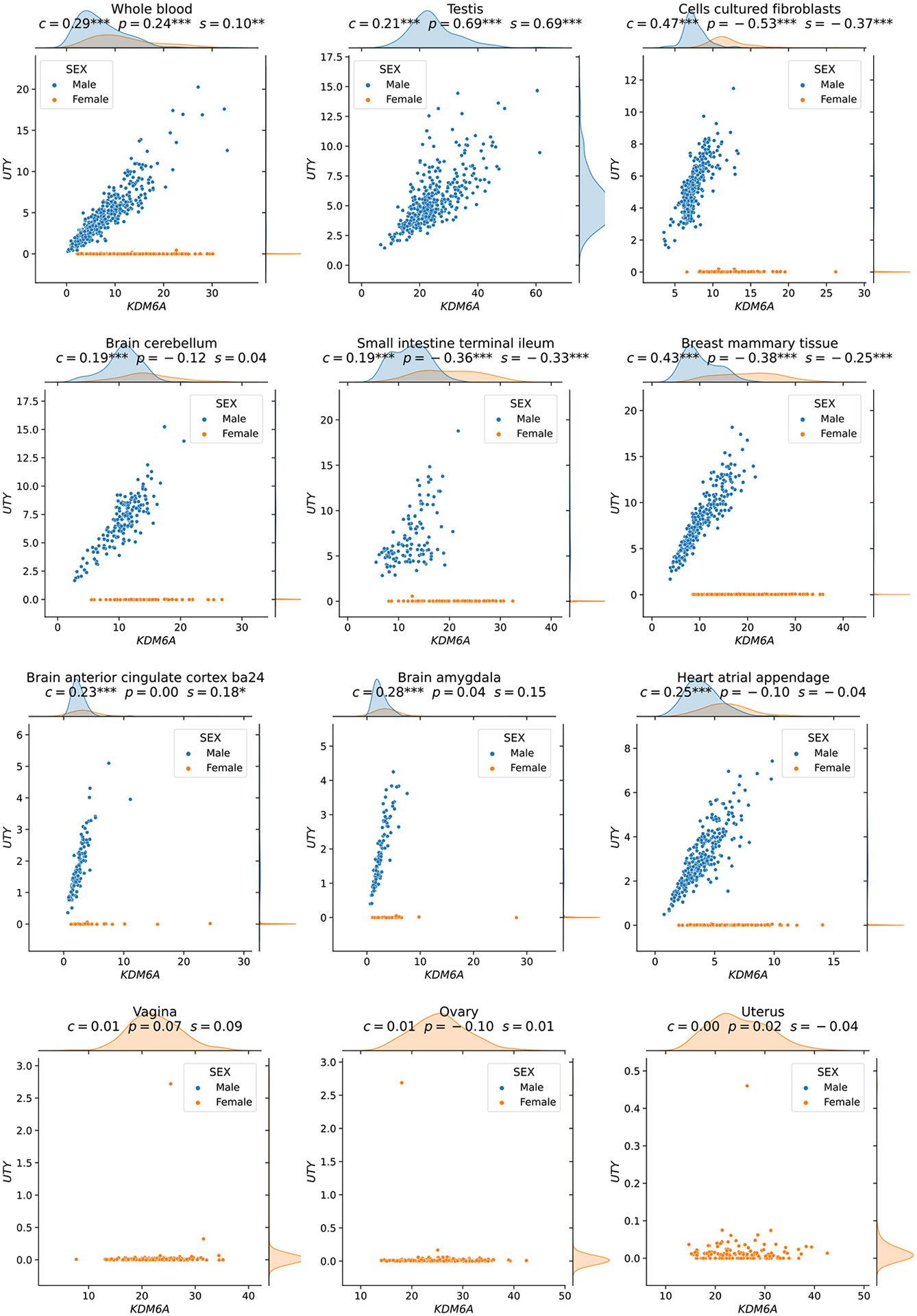
The expression levels of KDM6A and UTY display sex-specific associations across GTEx tissues CCC captures this nonlinear relationship in all GTEx tissues (nine examples are shown in the first three rows), except in female-specific organs (last row).

The first two gene pairs at the top of Figure 3B (*IFNG-SDS*, with high CCC and Spearman, and low Pearson; *PRSS36-CCL18*, with high CCC and low Pearson) appear to follow a non-coexistence relationship: in samples where one of the genes is highly expressed, the other is slightly activated, suggesting a potentially inhibiting effect. The following four gene pairs (*UTY-KDM6A, DDX3Y-KDM6A, RASSF2-CYTIP*, and *AC068580.6-KLHL21*) follow patterns combining either two linear or one linear and one independent relationship. In particular, genes *UTY-KDM6A* (paralogs) and *DDX3Y-KDM6A* show a nonlinear relationship where a subset of samples follows a robust linear pattern and another subset has a constant (independent) expression of one gene. The relationships in these two gene pairs are explained by sex differences in expression: *UTY* and *DDX3Y* are in chromosome Y (Yq11), whereas *KDM6A* is in chromosome X (Xp11), and therefore samples with a linear pattern are males, whereas those with no expression for *UTY* or *DDX3Y* are females. Furthermore, for this sex-specific gene pair pattern, CCC yields a statistically significant coefficient value in 45 out of 47 tissues in GTEx, except for female-specific organs (Figures 4 and S1; see “Correlations for *UTY - KDM6A* and *DDX3Y - KDM6A* in all GTEx tissues” in the key resources table). The gene pair *RASSF2-CYTIP* was replicated in an independent dataset as we explain later. Even though we have not found a biological explanation for gene pair *AC068580.6-KLHL21* (there is limited information about *AC068580.6*, ENSG00000235027, a long non-coding RNA), its strong nonlinear connection with *KLHL21* (linked with some cancers^37^) is robustly captured by CCC only. Notably, these four gene pairs contain strong linear relationships, and CCC is the only coefficient able to consistently capture these nonlinear patterns across a variety of tissues with a statistically significant and high correlation value. Pearson and Spearman show a statistically significant correlation value for some of these gene pairs, although these values are low and would very likely not be prioritized for further research. In addition, these two linear-only coefficients are unable to robustly capture the same pattern in other tissues (Figures 4 and S1; see “Correlations for *UTY - KDM6A* and *DDX3Y - KDM6A* in all GTEx tissues” in the key resources table). For instance, although the three coefficients are statistically significant in whole blood for the gene pair *UTY-KDM6A*, Pearson and Spearman fail to capture the same pattern in the brain cerebellum, and in many cases, such as small intestine, the sign of the coefficient is negative despite the strong positive linear correlation among male samples (Figure 4).

Finally, the last two gene pairs in Figure 3B are highly ranked by Pearson but not by CCC or Spearman. Although all coefficients are significant for the gene pair *MYOZ1-TNNI2*, the low CCC (c=0.03) and moderate Spearman (s=0.28) contrast with Pearson’s (*p* = 0:97), suggesting a statistically significant but very weak linear relationship. The high and statistically significant Pearson value for *SCGB3A1-C19orf33* seems to be driven by outliers.

### Replication of gene associations using tissue-specific gene networks from GIANT

We sought to systematically analyze discrepant scores to assess whether associations were replicated in other datasets besides GTEx. This is challenging and prone to bias because linear-only correlation coefficients are usually used in gene co-expression analyses. Therefore, we used 144 tissue-specific gene networks from the GIANT project,^7,38^ where nodes represent genes and each edge a functional relationship weighted with a probability of interaction between two genes (see STAR Methods). The version of GIANT used in this study did not include GTEx samples,^39^ making it an ideal case for replication. These networks were built from expression and different interaction measurements, including protein interaction, transcription factor regulation, chemical/genetic perturbations, and microRNA target profiles from the Molecular Signatures Database (MSigDB^40^). We reasoned that statistically significant and highly ranked gene pairs using three different coefficients in a single tissue (whole blood in GTEx, Figure 3) that represented real patterns should often replicate in a corresponding tissue or related cell lineage using the multi-cell type functional interaction networks in GIANT. In addition to predicting a network with interactions for a pair of genes, the GIANT web application can also automatically detect a relevant tissue or cell type where genes are predicted to be specifically expressed (the approach uses a machine learning method introduced in Ju et al.^41^ and described in STAR Methods).

**Figure 5.**
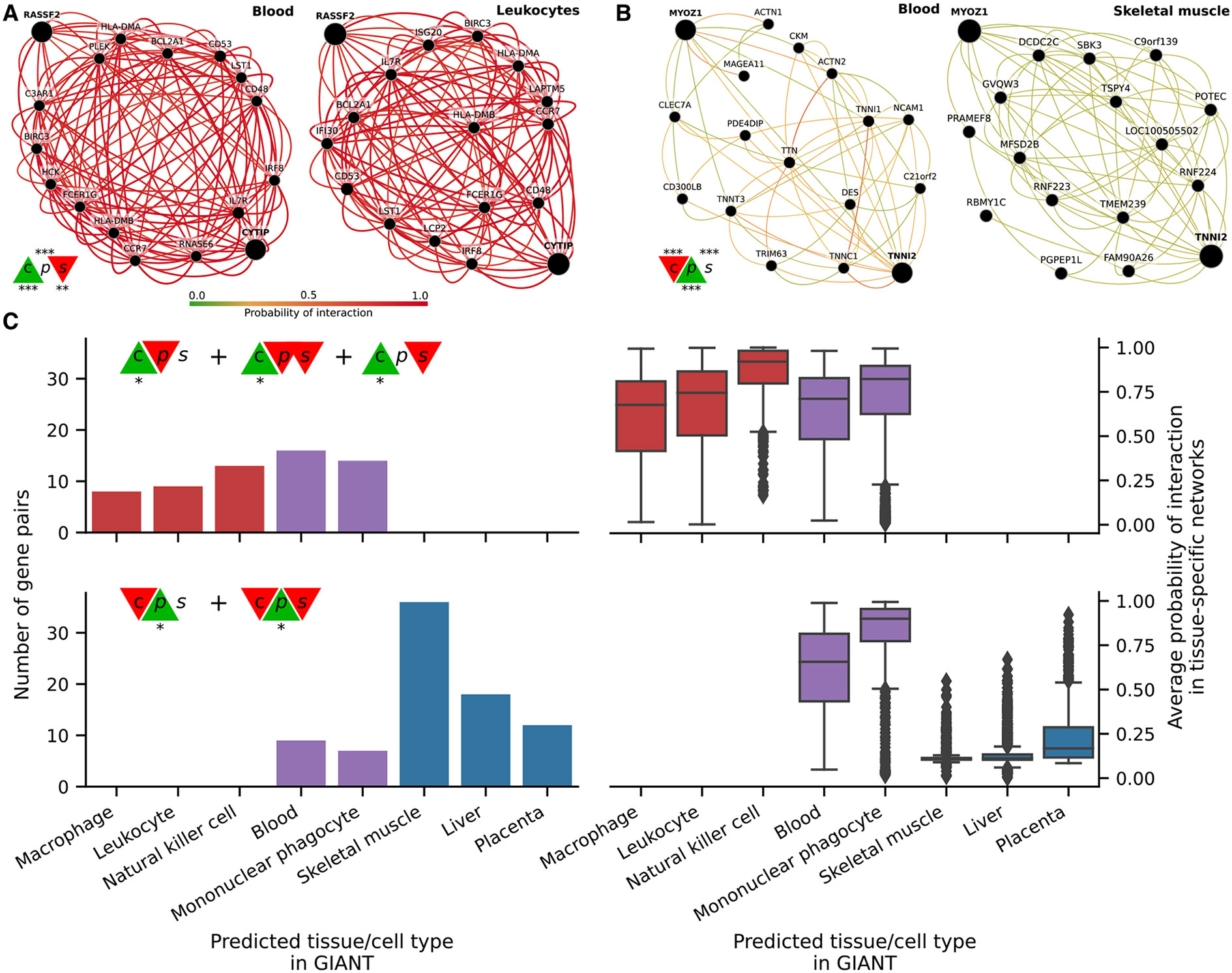
Analysis of GIANT tissue-specific predicted networks for gene pairs prioritized by correlation coefficients (A and B) Two gene pairs prioritized by correlation coefficients (from Figure 3B) with their predicted networks in blood (left) and an automatically selected tissue/cell type (right) using the method described in Zhang et al.^41^ A node represents a gene, and an edge represents the probability that two genes are part of the same biological process in a specific cell type. A maximum of 15 genes are shown for each network. The GIANT web application automatically determined a minimum interaction confidence (edges’ weights) to be shown. These networks can be analyzed online using the following links: *RASSF2-CYTIP*,^42^ *MYOZ1-TNNI2*.^43^ (C) Summary of predicted tissue/cell type networks for gene pairs exclusively prioritized by CCC and Pearson (FDR < 0:05). The first row combines all gene pairs where CCC is high, and Pearson and Spearman are not. The second row combines all gene pairs where Pearson is high, and CCC and Spearman are not. Bar plots (left) show the number of gene pairs for each predicted tissue/cell type. Boxplots (right) show the average probability of interaction between genes in these predicted tissue-specific networks. Red indicates CCC-only tissues/cell types, blue is Pearson-only, and purple is shared.

As an example of our evaluation procedure, we obtained the networks in blood and the automatically predicted cell type for gene pairs *RASSF2-CYTIP* (strong nonlinear pattern and CCC high, Figure 5A) and *MYOZ1-TNNI2* (weak linear pattern and Pearson high, Figure 5B). In addition to the gene pair, the networks include other genes connected according to their probability of interaction (up to 15 additional genes are shown), which allows estimating whether genes are part of the same tissue-specific biological process. Two large black nodes in each network’s top-left and bottom-right corners represent our gene pairs. A green edge means a close-to-zero probability of interaction, whereas a red edge represents a strong predicted relationship between the two genes. In this example, genes *RASSF2* and *CYTIP* (Figure 5A), with a high CCC value (c=0.20, above the 73th percentile) and low Pearson and Spearman (*p* = 0:16 and s=0.11, below the 38th and 17th percentiles, respectively), were both strongly connected to the blood network, with interaction scores of at least 0.69 and an average of 0.77 and 0.85, respectively (Table S1). The autodetected cell type for this pair was leukocytes, and interaction scores were similar to the blood network (Table S1). However, genes *MYOZ1* and *TNNI2*, with a very high Pearson value (*p* = 0:97), moderate Spearman (s=0.28), and very low CCC (c=0.03), were predicted to belong to much less cohesive networks (Figure 5B), with average interaction scores of 0.17 and 0.22 with the rest of the genes, respectively. Additionally, the autodetected cell type (skeletal muscle) is not related to blood or one of its cell lineages. These preliminary results suggested that CCC might be capturing blood-specific patterns missed by the other coefficients.

We next performed a systematic evaluation using the top 100 discrepant gene pairs between CCC and the other two coefficients. For each gene pair prioritized in GTEx (whole blood), we autodetected a relevant cell type using GIANT to assess whether genes were predicted to be specifically expressed in a blood-relevant cell lineage. For this, we used the top five most commonly autodetected cell types for each coefficient and assessed connectivity in the resulting networks (see STAR Methods). The top 5 predicted cell types for gene pairs highly ranked by CCC and not by the rest were all blood-specific (Figure 5C, top left), including macrophage, leukocyte, natural killer cell, blood, and mononuclear phagocyte. The average probability of interaction between genes in these CCC-ranked networks was significantly higher than the other coefficients (Figure 5C, top right), with all medians larger than 67% and first quartiles above 41% across predicted cell types. By contrast, most Pearson’s gene pairs were predicted to be specific to tissues unrelated to blood (Figure 5C, bottom left), with skeletal muscle being the most commonly predicted tissue. The interaction probabilities in these Pearson-ranked networks were also generally lower than in CCC, except for blood-specific gene pairs (Figure 5C, bottom right).

The associations exclusively detected by CCC in whole blood from GTEx were more strongly replicated in these independent networks that incorporated multiple data modalities. CCC-ranked gene pairs not only had higher probabilities of belonging to the same biological process but were also predicted to be specifically expressed in blood cell lineages. Conversely, most Pearson-ranked gene pairs were not predicted to be blood-specific, and their interaction probabilities were relatively much lower. This lack of replication in GIANT suggests that top Pearson-exclusive-ranked gene pairs in GTEx might be driven mainly by outliers, which is consistent with our earlier observations of outlier-driven associations (Figure 3B).

### CCC and the MIC

#### Conceptual and statistical differences

The CCC and the MIC^23^ are measures designed to capture nonlinear relationships between variables. While they share certain similarities, there are also notable differences between them.

Conceptually, CCC is grounded in clustering input data using each variable separately. This process effectively transforms each variable into a set of partitions, each containing a different number of clusters. The CCC then quantifies the correlation between variables by assessing the similarity of these partitions. This allows to process of various types of variables, including both numerical and categorical variables, even when the categories are nominal (i.e., they lack intrinsic order), as explained in STAR Methods. MIC, however, is specifically designed for numerical variables. Additionally, in theory, CCC should also support correlating variables with different dimensions. For onedimensional variables (such as genes), CCC obtains partitions using a quantile-based approach. For multidimensional variables, CCC could potentially use a standard clustering algorithm (such as k-means) to obtain partitions.

Now, consider two variables with n data points on a scatterplot. We can overlay a grid on this scatterplot with x columns and y rows, where each cell of this grid contains a portion of the data points, thereby defining a bivariate probability distribution. The MIC algorithm seeks an optimal grid configuration that maximizes the ratio of mutual information to logmin{x,y}, subject to the constraint that $xy<n^{0.6}$. This normalization process using logmin{x,y} scales the MIC score between zero and one. The CCC, as defined in STAR Methods, also generates a symmetric, normalized score between zero and one. However, unlike MIC, which utilizes normalized mutual information, CCC employs the ARI. The ARI has an advantageous property: it consistently returns a baseline (zero) for independently drawn partitions, irrespective of the number of clusters (see Figure S2). This property is not inherent in mutual information, which can produce varied values for independent variables if the grid dimensions vary. MIC mitigates this by limiting the grid size with the constraint $xy<n^{0.6}$, which could also limit its ability to detect complex relationships.

Both CCC and MIC involve binning the input data vectors, aiming to maximize the mutual information and the ARI, respectively. However, their approaches differ in complexity and execution. MIC utilizes a sophisticated dynamic programming algorithm to identify the optimal grid. By contrast, CCC employs a more straightforward and faster method, partitioning the data points separately using the two vectors. While CCC might benefit from adopting MIC’s more complex grid search approach, it remains uncertain if MIC could maintain its performance using CCC’s simpler partitioning strategy.

Regarding their parameters, CCC’s $k_{max}$ (maximum number of clusters) and MIC’s B(n) (maximum grid size) serve similar purposes. They control both the complexity of the patterns detected and the computational time. For example, as illustrated in Figure 1 (Anscombe I and III), a $k_{max}$ of 2 is adequate for identifying linear patterns but insufficient for more complex patterns like quadratic or two-lines patterns. A similar principle applies to MIC’s B(n). However, a critical distinction exists between the two: the constant baseline property of ARIs ensures that CCC returns a value close to zero for independent variables, regardless of $k_{max}$. By contrast, MIC may produce non-zero scores for independent data if B(n) is set too high, as discussed in Section 2.2.1 of the supplementary material in Reshef et al.^23^ The authors of MIC suggest that a value of $B(n)=n^{0.6}$ is generally effective in practice.

#### Comparison in gene expression data

**Figure 6.**
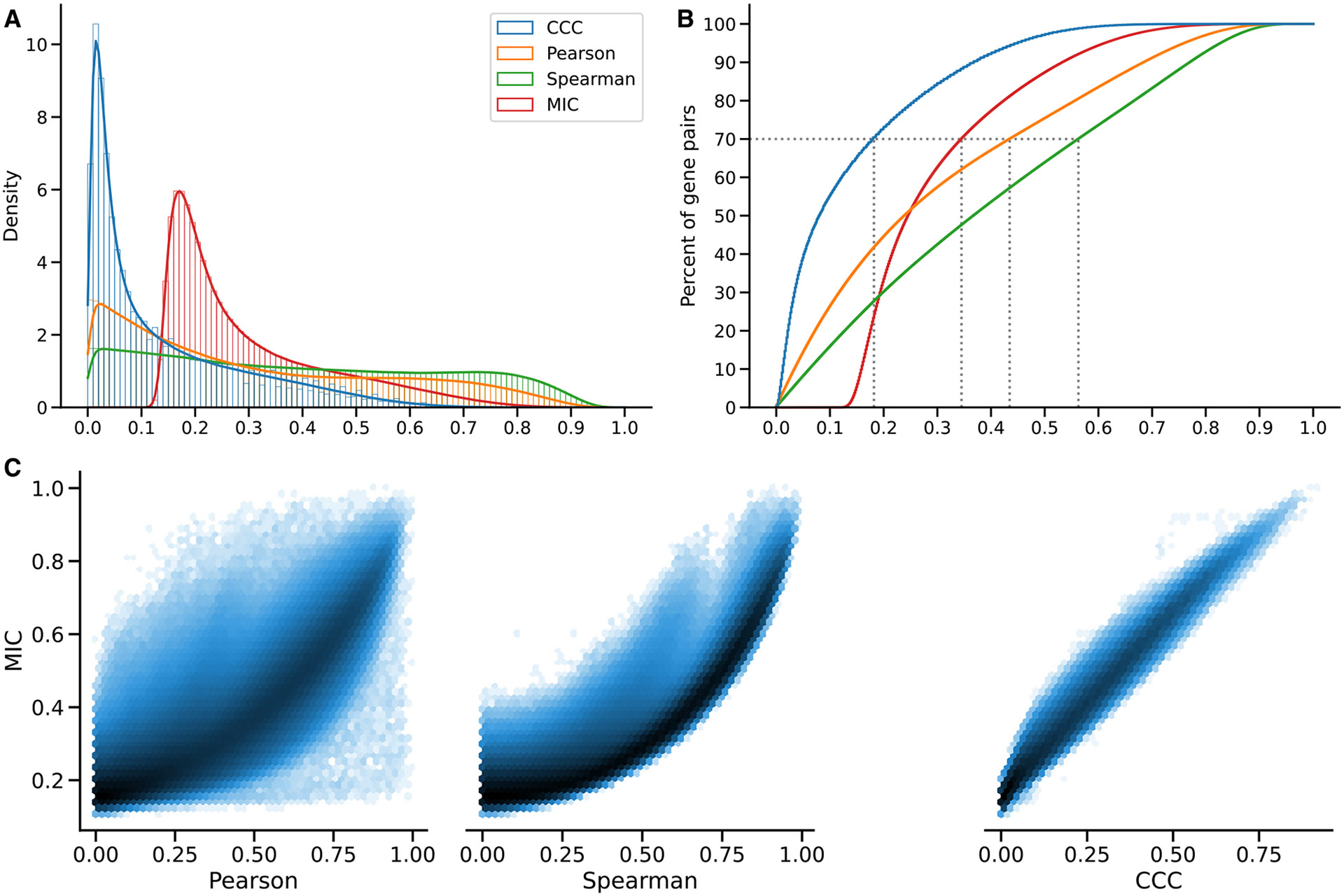
Distribution of MIC values on gene expression (GTEx v8, whole blood) and comparison with other methods (A) Histogram of coefficient values. (B) Corresponding cumulative histogram. The dotted line maps the coefficient value that accumulates 70% of gene pairs. (C) 2D histogram plot with hexagonal bins between all coefficients, where a logarithmic scale was used to color each hexagon.

We compared all the coefficients in this study with MIC, a popular nonlinear method that can find complex relationships in data, although it is very computationally intensive.^25^ We ran MICe (see STAR Methods) on all possible pairwise comparisons of our 5,000 highly variable genes from whole blood in GTEx v8. Then we performed the analysis on the distribution of coefficients (the same as in the main text), shown in Figure 6. We verified that CCC and MIC behave similarly in this dataset, with essentially the same distribution but only shifted. Figure 6C shows that these two coefficients relate almost linearly, and both compare very similarly with Pearson and Spearman.

### Computational complexity of coefficients

**Figure 7.**
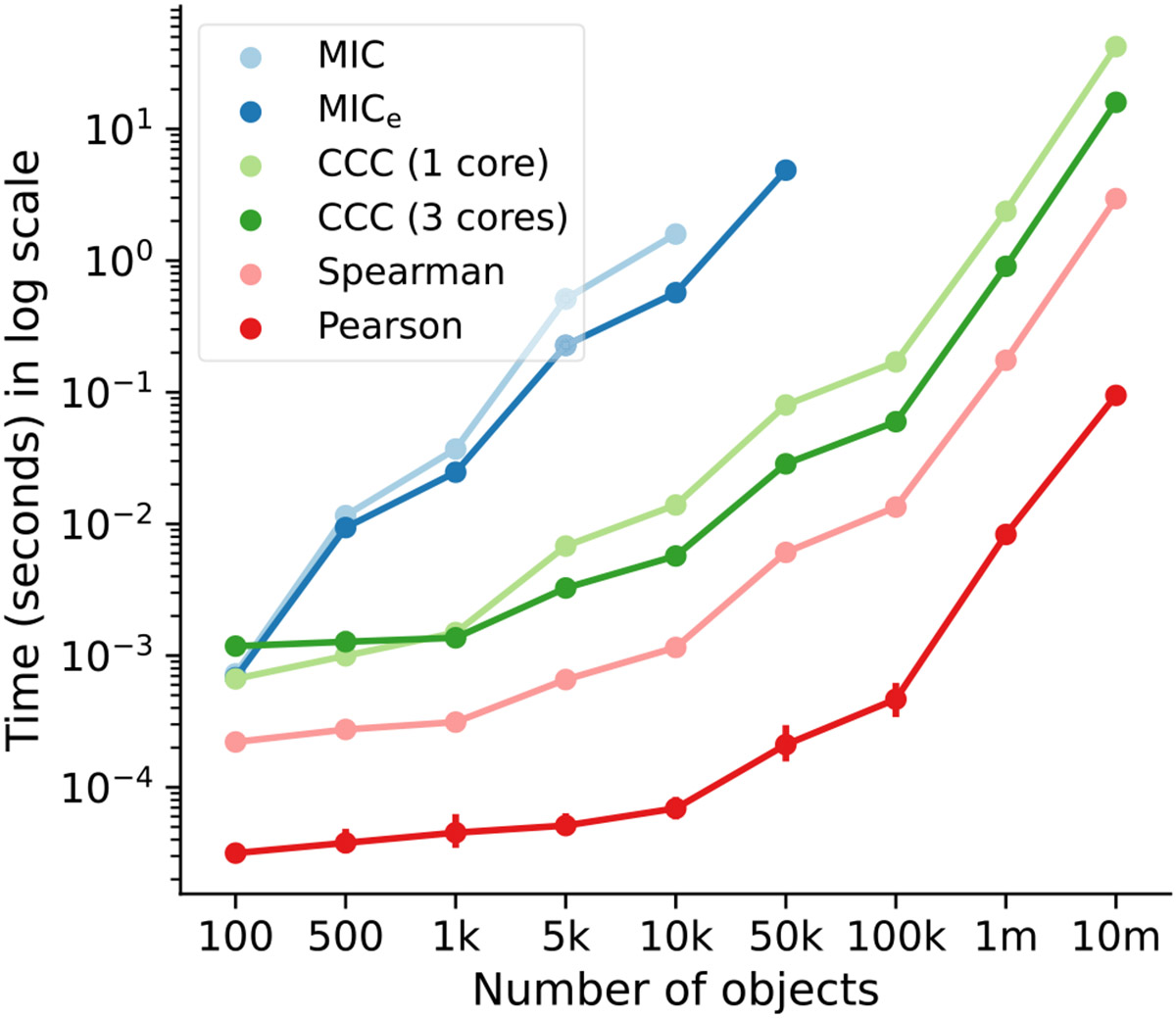
Computational complexity of all correlation coefficients on simulated data We simulated variables/features with varying data sizes (from 100 to a million, x axis). The plot shows the average time in seconds (log-scale) taken for each coefficient on ten repetitions (1,000 repetitions were performed for data size 100). CCC was run using 1 and 3 CPU cores. MIC and MICe did not finish running in a reasonable amount of time for data sizes of 10,000 and 100,000, respectively.

We also compared CCC with the other coefficients in terms of computational complexity. Although CCC and MIC might identify similar gene pairs in gene expression data (see here), the use of MIC in large datasets remains limited due to its very long computation time, despite some methodological/implementation improvements.^25,27,44-46^ The original MIC implementation uses ApproxMaxMI, a computationally demanding heuristic estimator.^23^ Recently, a more efficient implementation called MICe was proposed.^47^ These two MIC estimators are provided by the minepy package,^44^ a C implementation available for Python. We compared all these coefficients in terms of computation time on randomly generated variables of different sizes, which simulates a scenario of gene expression data with different numbers of conditions. Differently from the rest, CCC allows us to easily parallelize the computation of a single gene pair (see STAR Methods), so we also tested the cases using 1 and 3 central processing unit (CPU) cores. Figure 7 shows the time in seconds in log scale.

As we already expected, Pearson and Spearman were the fastest, given that they only need to compute basic summary statistics from the data. For example, Pearson is three orders of magnitude faster than CCC. Among the nonlinear coefficients, CCC was faster than the two MIC variations (up to two orders of magnitude), with the only exception in very small data sizes. The difference is important because both MIC variants were implemented in C^51^, a high-performance programming language, whereas CCC was implemented in Python (optimized with Numba). For a data size of a million, the multi-core CCC was twice as fast as the single-core CCC.

## DISCUSSION

We introduce the CCC, an efficient, not-only-linear clustering-based statistic. Applying CCC to GTEx v8 revealed that it was robust to outliers and detected linear relationships as well as complex and biologically meaningful patterns that standard coefficients missed. In particular, CCC alone detected gene pairs with complex nonlinear patterns from the sex chromosomes, highlighting the way that not-only-linear coefficients can play in capturing sex-specific differences. The ability to capture these nonlinear patterns, however, extends beyond sex differences: it provides a powerful approach to detect potentially complex relationships where a subset of samples or conditions are explained by other factors (such as differences between health and disease). We found that the top CCC-ranked gene pairs in whole blood from GTEx were replicated in independent tissue-specific networks trained from multiple data types and attributed to cell lineages from blood, even though CCC did not have access to any cell lineage-specific information. This suggests that CCC can disentangle intricate cell lineage-specific transcriptional patterns missed by linear-only coefficients. In addition to capturing nonlinear patterns, the CCC was more similar to Spearman than Pearson, highlighting their shared robustness to outliers. The CCC results were concordant with MIC, but much faster to compute and thus practical for large datasets. Another advantage over MIC and standard coefficients is that CCC can also process categorical variables together with numerical values. CCC is conceptually easy to interpret and has a single parameter that controls the maximum complexity of the detected relationships while also balancing compute time.

Datasets such as Anscombe or “Datasaurus” highlight the value of visualization instead of relying on simple data summaries. While visual analysis is helpful, for many datasets, examining each possible relationship is infeasible, and this is where more sophisticated and robust correlation coefficients are necessary. Advanced yet interpretable coefficients like CCC can focus human interpretation on patterns that are more likely to reflect real biology. The complexity of these patterns might reflect heterogeneity in samples that mask clear relationships between variables. For example, genes *UTY-KDM6A* (from sex chromosomes), detected by CCC, have a strong linear relationship but only in a subset of samples (males), which was not captured by linear-only coefficients. This example, in particular, highlights the importance of considering sex as a biological variable (SABV)^48^ to avoid overlooking important differences between men and women, for instance, in disease manifestations.^49,50^ More generally, not-only-linear correlation coefficients that support categorical variables like CCC could identify significant differences between variables (such as genes) that are explained by a third factor (beyond sex differences) that would be entirely missed by linear-only coefficients.

It is well known that biomedical research is biased toward a small fraction of human genes.^51,52^ Some genes highlighted in CCC-ranked pairs (Figure 3B), such as *SDS* (12q24) or *PRSS36* (16p11), were previously found to be the focus of fewer than expected publications.^53^ It is possible that the widespread use of linear coefficients may bias researchers away from genes with complex co-expression patterns. A beyond-linear gene coexpression analysis on large compendia might shed light on the function of understudied genes. For example, genes *KLHL21* (1p36) and *AC068580.6* (a long non-coding RNA gene in 11p15) have a high CCC value and are missed by the other coefficients. *KLHL21* was suggested as a potential therapeutic target for hepatocellular carcinoma^37^ and other cancers.^54,55^ Its nonlinear correlation with *AC068580.6* might unveil other important players in cancer initiation or progression, potentially in subsets of samples with specific characteristics (as suggested in Figure 3B).

Not-only-linear correlation coefficients might also be helpful in the field of genetic studies. In this context, GWASs have been successful in understanding the molecular basis of common diseases by estimating the association between genotype and phenotype.^56^ However, the estimated effect sizes of genes identified with GWASs are generally modest, and they explain only a fraction of the phenotype variance, hampering the clinical translation of these findings.^57^ Recent theories, like the omnigenic model for complex traits,^16,17^ argue that these observations are explained by highly interconnected gene regulatory networks, with some core genes having a more direct effect on the phenotype than others. Using this omnigenic perspective, we and others^19,20,22^ have shown that integrating gene co-expression networks with genetic studies could potentially identify core genes that are missed by linear-only models alone like GWASs. Our results suggest that building these networks with the latest approaches^58^ and advanced and efficient correlation coefficients could better estimate gene co-expression profiles and thus more accurately identify these core genes. Approaches like CCC could play a significant role in the precision medicine field by providing the computational tools to focus on more promising genes representing potentially better candidate drug targets.

Our analyses have some limitations. We worked on a sample with the top variable genes in a single tissue from GTEx to keep computation time feasible. Although CCC is much faster than MIC, Pearson and Spearman are still the most computationally efficient since they only rely on simple data statistics. Our results, however, reveal the advantages of using more advanced coefficients like CCC for detecting and studying more intricate molecular mechanisms that are replicated in independent datasets. The application of CCC on larger compendia, such as recount3^11^ with thousands of heterogeneous samples across different conditions, can reveal other potentially meaningful gene interactions. We compute p values using computationally intensive permutation tests; in the future, we plan to explore efficient permutation approaches such as those based on extreme value theory.^59^ The single parameter of CCC, $k_{max}$, controls the maximum complexity of patterns found and also impacts the compute time. Our analysis suggested that $k_{max}=10$ was sufficient to identify both linear and more complex patterns in gene expression. A more comprehensive analysis of optimal values for this parameter could provide insights into how to adjust it for different applications or data types. Finally, computing the correlation between a gene pair represents only the first step of the analysis. Controlling for known confounders, integrating with other data types, and replicating in independent datasets are some of the other important steps to ensure the biological relevance of the detected patterns.

While linear and rank-based correlation coefficients are exceptionally fast to calculate, not all relevant patterns in biological datasets are linear. For example, patterns associated with SABV are not apparent to the linear-only coefficients that we evaluated but are revealed by not-only-linear methods. Beyond sex differences, being able to use a method that inherently identifies patterns driven by other factors is likely to be desirable. Not-only-linear coefficients can also disentangle intricate yet relevant patterns from expression data alone that were replicated in models integrating different data modalities. CCC, in particular, is highly parallelizable, and we anticipate efficient GPU-based implementations that could make it even faster. The CCC is an efficient, next-generation correlation coefficient that is highly effective in transcriptome analyses and potentially useful in a broad range of other domains.

## STAR★METHODS

### METHOD DETAILS

#### Data preprocessing

We downloaded gene expression data from GTEx v8 (https://gtexportal.org/) for all tissues, normalized using TPM (transcripts per million), and focused our primary analysis on whole blood, which has a good sample size (755). We selected the top 5,000 genes from whole blood with the largest variance after standardizing with log(x+1) to avoid a bias toward highly expressed genes. We then computed Pearson,^60,61^ Spearman,^60,61^ the Maximal Information Coefficient (MIC)^47^ and CCC on these 5,000 genes across all 755 samples, generating a pairwise similarity matrix of size 5,000 x 5,000.

#### The Clustermatch Correlation Coefficient (CCC)

##### Definitions

**Definition 1.1.** Given a data vector $x=(x_{1},x_{2},\ldots ,x_{n})\in R^{n}$ then define

$$\pi_{\ell }=\{i|\rho_{\ell }<x_{i}\leq \rho_{\ell +1}\},\forall \ell \in [1,k]$$

as a *partition* of the n objects of x into ∣π∣=k clusters, where ρ is a set of k+1 cutpoints (e.g., quantiles) that define the clusters, with $\rho_{1}=min(x)$ and $\rho_{k+1}=max(x)$. If x is a categorical vector with no intrinsic ordering, then a partition is defined as

$$\pi_{\ell }=\{i|x_{i}=C_{\ell }\},\forall \ell \in [1,|C|]$$

where $C=\{c_{1},c_{2},\ldots ,c_{m}\}$ is a set of unique values in x corresponding to the m=∣C∣ categorical values that define the clusters. **Definition 1.2.** Given two partitions π and $\pi^{\prime }$ of n objects, the *adjusted Rand Index (ARI)*^29^ is given by

$$ARI(\pi ,\pi^{\prime })=\frac{2(n_{0}n_{1}-n_{2}n_{3})}{\left(n_{0}+n_{2})(n_{2}+n_{1})+(n_{0}+n_{3})(n_{3}+n_{1}\right)},$$

where $n_{0}$ is the number of object pairs that are in the same cluster in both partitions π and $\pi^{\prime }$, $n_{1}$ is the number of object pairs that are in different clusters, $n_{2}$ is the number of object pairs that are in the same cluster in π but in different clusters in $\pi^{\prime }$, and $n_{3}$ is the number of object pairs that are in different clusters in π but in the same cluster in $\pi^{\prime }$. Intuitively, $n_{0}+n_{1}$ reflects the number of object pairs where both partitions agree, and $n_{2}+n_{3}$ are those in which they disagree.

**Definition 1.3.** The *Clustermatch Correlation Coefficient (CCC)* between x and y is defined as the maximum ARI between all possible partitions of x and y

CCC(x,y)=max{{0,maxπj∈Πxπi∈Πy{ARI(πj,πi)}},∀∣π∣∈[2,kmax]

where $\Pi^{x}$ is a set of partitions derived from x, $\Pi^{y}$ is a set of partitions derived from y, and $k_{max}$ specifies the maximum number of clusters allowed. The ARI has an upper bound of 1 (achieved when both partitions are identical), and although it does not have a well-defined lower bound, values equal or less than zero are achieved when partitions are independent. Therefore, CCC(x,y)∈[0,1]. In the special case where all n objects in either x or y have the same value, the CCC is undefined.

The CCC has the following basic properties derived from the ARI: 1) symmetry, since $ARI(\pi ,\pi^{\prime })=ARI(\pi^{\prime },\pi )$ 2) normalization, since it takes a minimum value of zero and a maximum of one since ARI(π,π)=1 3) constant baseline, since the ARI is adjusted-for-chance,^29^ it returns a value close to zero for independently drawn partitions, and this also holds when partitions have different number of clusters.^62^ This is an important property, since CCC compares partitions with different numbers of clusters, and relationships between two variables (such as linear or quadratic) might be better represented with different numbers of clusters as shown in Figure 1.

##### The maximum number of clusters $k_{max}$

The parameter $k_{max}$ is the maximum number of clusters k allowed for any partition derived from x or y. On one hand, note that the same value of k might not be the right one to find a relationship between any two variables. For instance, in the quadratic example in Figure 1, CCC returns a value of 0.36 (grouping objects in four clusters using one variable and two using the other). If we used only two clusters instead, CCC would return a similarity value of 0.02. On the other hand, computational time increases quadratically with $k_{max}$. In addition, it is important to note that given the constant baseline property of the ARI, the CCC returns a value close to zero for independent variables regardless of the value of $k_{max}$. As shown in Figure S2, this holds even for very large values of $k_{max}$, approaching the number of objects n. Note that as $k_{max}$ approaches n, the number of singleton clusters (i.e., clusters with only one object) increases, which would not be useful for finding relationships between variables. Therefore, given the constant baseline property, $k_{max}$ only represents a tradeoff between the ability to capture complex patterns and the computational cost, with random/independent variables having a CCC value close to zero regardless of the value of $k_{max}$; we found that $k_{max}=10$ works well in practice, and it was used as the default maximum number of clusters across all our analyses.

##### Statistical significance

Our null hypothesis is that the variables represented by x and y are independent. To compute a *P*-value, we perform a set of permutations of values in y and compute the CCC between x and each permuted vector. The *P*-value is the proportion of CCC values using the permuted data that are greater than or equal to the CCC value between x and y. We used 1 million permutations in all our analyses, and we adjusted the *P*-values using the Benjamini and Hochberg procedure^36^ to control the false discovery rate (FDR); given the computational cost, we computed a *P*-value only for gene pairs from the “Disagreements” group in Figure 3, which contains gene pairs ranked differently by the correlation coefficients.

##### Strength of linear correlation

Figure 4 shows the relationships between *UTY* (chromosome Y) and *KDM6A* (chromosome X) across tissues in GTEx. For this gene pair, CCC can find a complex pattern where a subset of samples (males) follows a clear linear relationship, and there is no relationship in the rest of the samples (females). As it can be seen, there is a difference in the strength of the linear correlation between male samples across different tissues. For example, in brain cerebellum, the linear correlation among male samples is stronger than in small intestine (terminal ileum). As shown in Figure S3A, this difference is reflected by all coefficients when only male samples are considered.

However, when we consider all samples (males and females), there is no longer a linear relationship between *UTY* and *KDM6A*. Therefore, while a subset of the data displays linear relationships, overall, it is no longer true that there is a linear correlation. CCC assumes that if two variables (genes in our case) are similar, the clustering of objects (samples) using each variable separately should match. As shown in Figure S3A with red lines, this clustering of samples and their matching can be seen for the gene pair *UTY / KDM6A*: when we only consider male samples, CCC finds clusterings in brain cerebellum with a larger matching than in small intestine because the linear strength differs. But when we consider all samples together (males and females, as shown in Figure S3B), the pattern is nonlinear, the distribution of all the data is different, and so are the clusterings found by CCC.

The effect of analyzing *all* the data (males and females) in this nonlinear pattern (Figure 4) is also clear in the negative sign of Pearson and Spearman coefficients in small intestine or even other tissues with a very strong and clear linear pattern among male samples such as breast mammary tissue. This case indicates that Pearson and Spearman, although statistically significant, are capturing the wrong pattern. Therefore, the fact that CCC yields a similar value (0.19) for these nonlinear patterns in brain cerebellum and small intestine (Figure S3B) reflects a similar clustering matching when considering all the samples. When applied only to the data with linear relationships of varying strength, CCC performs consistently with other coefficients.

##### Presence of substructure in the data

Consider a scenario where there are known and undesirable substructures in the data. In the example in Figure S4, we have simulated two distinct clusters (normally distributed) placed diagonally, horizontally, and vertically. The only case where the CCC is close to 1.0 (Diagonal, left) is when the clusterings/partitions of objects using each variable (x and y) match, which coincides with a linear pattern. In the other two cases (Horizontal and Vertical), clusterings of objects do not match, leading to a CCC value of zero. We note that MIC has the same behavior.

#### Tissue-specific network analyses using GIANT

We accessed tissue-specific gene networks of GIANT using both the web interface and web services provided by HumanBase.^38^ The GIANT version used in this study included 987 genome-scale datasets with approximately 38,000 conditions from around 14,000 publications. Details on how these networks were built are described in Greene et al.^7^ Briefly, tissue-specific gene networks were built using gene expression data (without GTEx samples^39^) from the NCBI’s Gene Expression Omnibus (GEO),^63^ protein-protein interaction (BioGRID,^64^ IntAct,^65^ MINT^66^ and MIPS^67^), transcription factor regulation using binding motifs from JASPAR,^68^ and chemical and genetic perturbations from MSigDB.^40^ Gene expression data were log-transformed, and the Pearson correlation was computed for each gene pair, normalized using the Fisher’s z transform, and z-scores discretized into different bins. Gold standards for tissue-specific functional relationships were built using expert curation and experimentally derived gene annotations from the Gene Ontology. Then, one naive Bayesian classifier (using C++ implementations from the Sleipnir library^69^) for each of the 144 tissues was trained using these gold standards. Finally, these classifiers were used to estimate the probability of tissue-specific interactions for each gene pair.

For each pair of genes prioritized in our study using GTEx, we used GIANT through HumanBase to obtain 1) a predicted gene network for blood (manually selected to match whole blood in GTEx) and 2) a gene network with an automatically predicted tissue using the method described in Ju et al.^41^ and provided by HumanBase web interfaces/services. Briefly, the tissue prediction approach trains a machine learning model using comprehensive transcriptional data with human-curated markers of different cell lineages (e.g., macrophages) as gold standards. Then, these models are used to predict other cell lineage-specific genes. In addition to reporting this predicted tissue or cell lineage, we computed the average probability of interaction between all genes in the network retrieved from GIANT. Following the default procedure used in GIANT, we included the top 15 genes with the highest probability of interaction with the queried gene pair for each network.

#### Maximal Information Coefficient (MIC)

We used the Python package minepy^44,70^ (version 1.2.5) to estimate the MIC coefficient. In GTEx v8 (whole blood), we used MICe (an improved implementation of the original MIC introduced in Reshef et al.^47^) with the default parameters alpha=0.6, c=15 and estimator=‘mic_e’. We used the pairwise_distances function from scikit-learn^60^ to parallelize the computation of MIC on GTEx. For our computational complexity analyses, we ran the original MIC (using parameter estimator=‘mic_approx’) and MICe (estimator=‘mic_e’).

## Supplementary Material

MMC2

MMC1

## Figures and Tables

**Table:** KEY RESOURCES TABLE

REAGENT or RESOURCE	SOURCE	IDENTIFIER
Deposited data		
GTEx v8	The GTEx Consortium^10^	https://gtexportal.org/
All pairwise gene correlations in GTEx whole blood	This paper	https://doi.org/10.5281/zenodo.10472273
Coefficients percentiles in GTEx whole blood	This paper	https://doi.org/10.5281/zenodo.10472273
Correlations for *UTY - KDM6A* and *DDX3Y - KDM6A* in all GTEx tissues	This paper	https://doi.org/10.5281/zenodo.10472273
Software and algorithms		
Python 3.9.16	Python Software Foundation	https://python.org/
scikit-learn-0.24.2	Pedregosa et al.^60^	https://github.com/scikit-learn/scikit-learn
SciPy	Virtanen et al.^61^	https://github.com/scipy/scipy
MIC	Reshef et al.^47^	https://github.com/minepy/minepy
GIANT	Greene et al.^7^	https://hb.flatironinstitute.org/
CCC	This paper	GitHub: https://github.com/greenelab/ccc or Zenodo: https://doi.org/10.5281/zenodo.13304247

## Box

**Box 1.** The CCC algorithm

Algorithm 1: CGC algorithm
1Functionget_partitions(v,kmax):23456789101112⌊Input:v:feature values onnobjectskmax:maximum number of clustersOutput:Π:a set of partitions overnobjectsifv∈Rn⌊fork←2tomin{kmax,n−1}do⌊ρ←(ρℓ∣Pr(vi<ρℓ)≤(ℓ−1)∕k),∀ℓ∈[1,k+1]πℓ←{i∣ρℓ<vi≤ρℓ+1},∀ℓ∈[1,k]Πk←π⌋⌋else⌊𝒞←{c1,c2,…,cm}(set ofmunique categorical values inv)πℓ←{i∣vi=𝒞ℓℓ∈[1,m]Πm←π⌋Π←{Πk∣∣Πk∣>1},∀kreturnΠ⌋1314Functionccc(x,y,kmax):15161718⌊Input:x:feature values onnobjectsy:feature values onnobjectskmax:maximum number of clustersOutput:c:correlation value forxandy(c∈[0,1])Πx=get_partitions(x,kmax)Πy=get_partitions(y,kmax)c←max{ARI{πj,πl)},∀πj∈Πx,πl∈Πyreturnmax(c,0)⌋

The main function of the algorithm, CCC, generates a set of partitions $\Pi^{x}$ for variable x (line 16) and another set of partitions $\Pi^{y}$ for variable y (line 17). Then, it computes the ARI between each partition $\pi_{j}\in \Pi^{x}$ and $\pi_{i}\in \Pi^{y}$ and gets the maximum (line 18), returning either this value or zero if this is negative (line 19).

Since CCC only needs a set of partitions to compute a correlation value, any type of variable that can be used to perform clustering is supported. If variable v is numerical (lines 2–6 in the get_partitions function), each partition π is generated using a set of quantiles ρ. For example, if k=2, then $\rho =(\rho_{1},\rho_{2},\rho_{3})$, where $\rho_{1}$ is the minimum value of v, $\rho_{2}$ is the median, and $\rho_{3}$ is the maximum value of v. Then, the first cluster $\pi_{1}$ contains all values of v that are less than or equal to $\rho_{2}$, and $\pi_{2}$ contains all values of v that are greater than $\rho_{2}$. If variable v is categorical (lines 8–11), we compute a single partition π with m=∣C∣ clusters, where $C=\{c_{1},c_{2},\ldots ,c_{m}\}$ is a set of unique categorical values in v. Therefore, all variable types are internally represented as partitions, and it is not necessary to access the original data values to compute the ARI. Consequently, numerical and categorical variables can be naturally integrated.

Our algorithm implementation uses $k_{max}=10$ as the default. This means that for a variable pair, 18 partitions are generated (9 for each variable, from k=2 to k=10), and 81 ARI comparisons are performed. Smaller values of $k_{max}$ can reduce computation time, although at the expense of missing more complex/general relationships. Our examples in Figure 1 suggest that using $k_{max}=2$ would force CCC to find linear-only patterns, which could be a valid use-case scenario where only this kind of relationship is desired. In addition, $k_{max}=2$ implies that only two partitions are generated and only one ARI comparison is performed.

As a final remark, generating partitions (lines 15 and 16) and computing their similarity (line 17) can be easily parallelized. We used three CPU cores in our analyses to speed up the computation of CCC, and this could be potentially extended to a large number of processors using a graphics processing unit (GPU).

## Highlights

- CCC is a not-only-linear, computationally efficient correlation coefficient
- CCC is a clustering-based statistic
- CCC reveals meaningful linear and nonlinear patterns missed by standard coefficients
- It can readily be applied to genome-scale data across different data types
