# Supplementary material for: An efficient, not-only-linear correlation coefficient based on clustering
Source: Cell Syst. Author manuscript; Available in PMC 2025 Mar 28. (PMC11951854; doi:10.1016/j.cels.2024.08.005)
Supplement: MMC1 [file NIHMS2023070-supplement-MMC1.pdf]

Cell Systems, Volume *15*

Supplemental information

An efficient, not-only-linear correlation coefficient based on clustering

Milton Pividori, Marylyn D. Ritchie, Diego H. Milone, and Casey S. Greene

Supplemental information

Figures

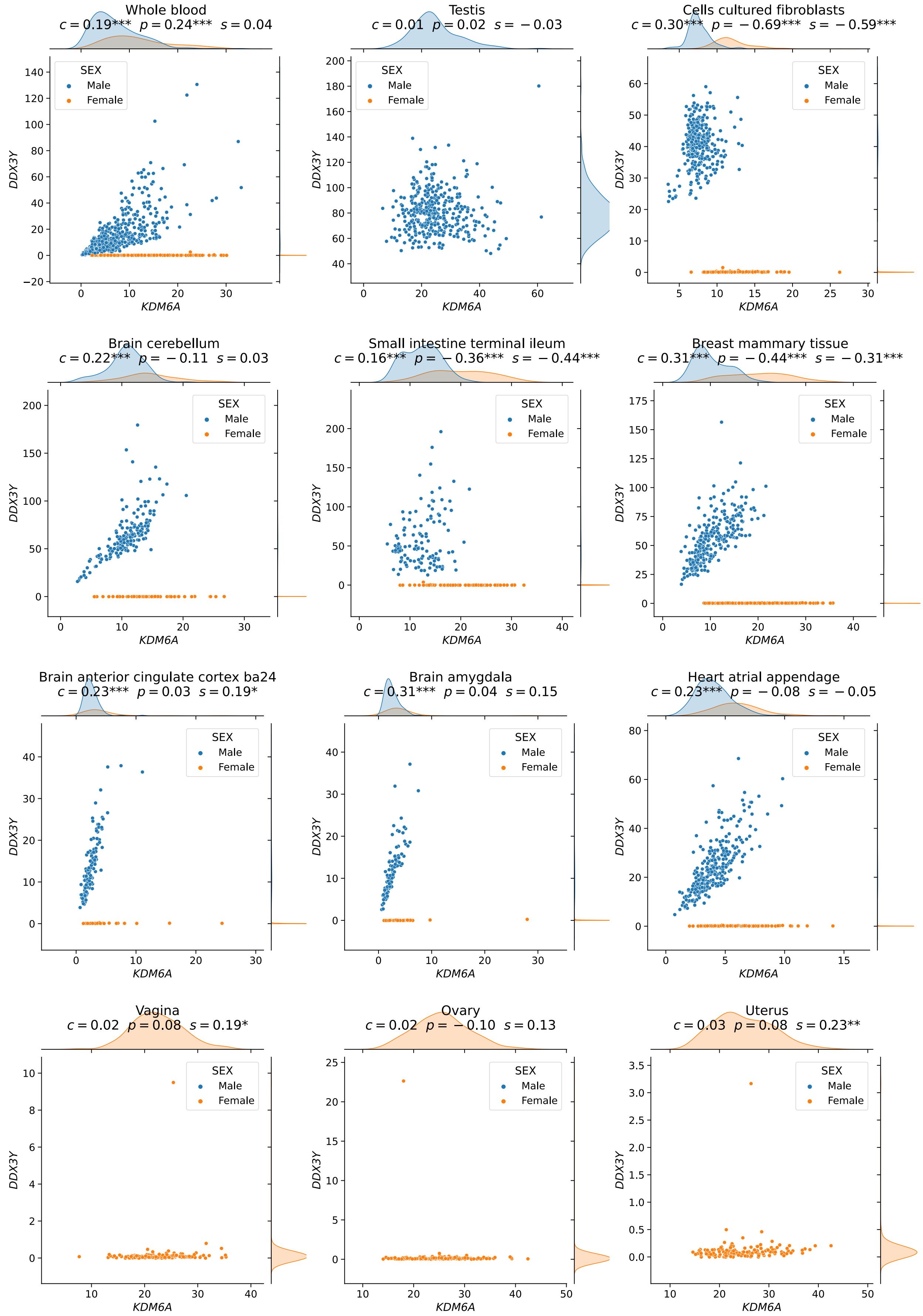

Figure S1: The expression levels of *KDM6A* and *DDX3Y* display sex-specific associations across GTEx tissues. CCC captures this nonlinear relationship in all GTEx tissues (nine examples are shown in the first three rows), except in female-specific organs (last row).

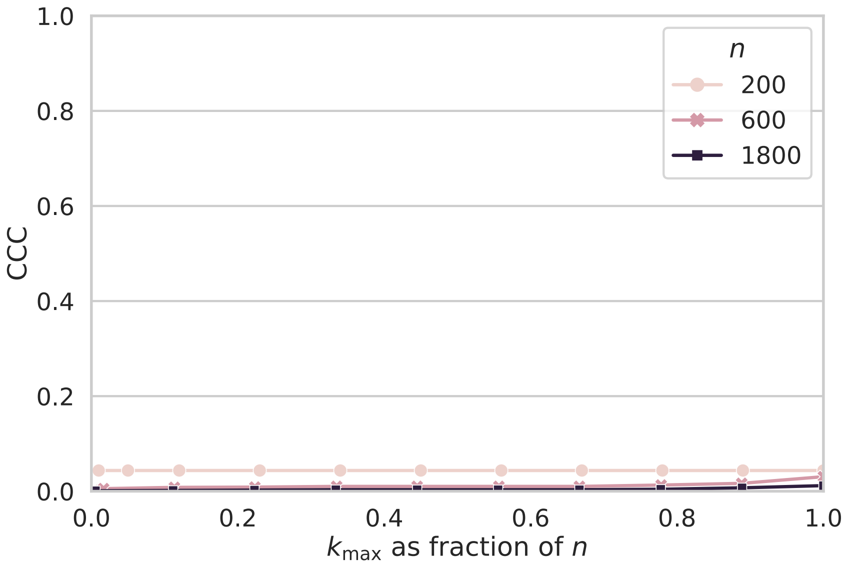

Figure S2: Constant baseline property: CCC values are close to zero for random/independent variables. The plot shows CCC values for normally distributed and independent variables with different sizes n and using different values for parameter $k_{\max}$ (maximum number of clusters).

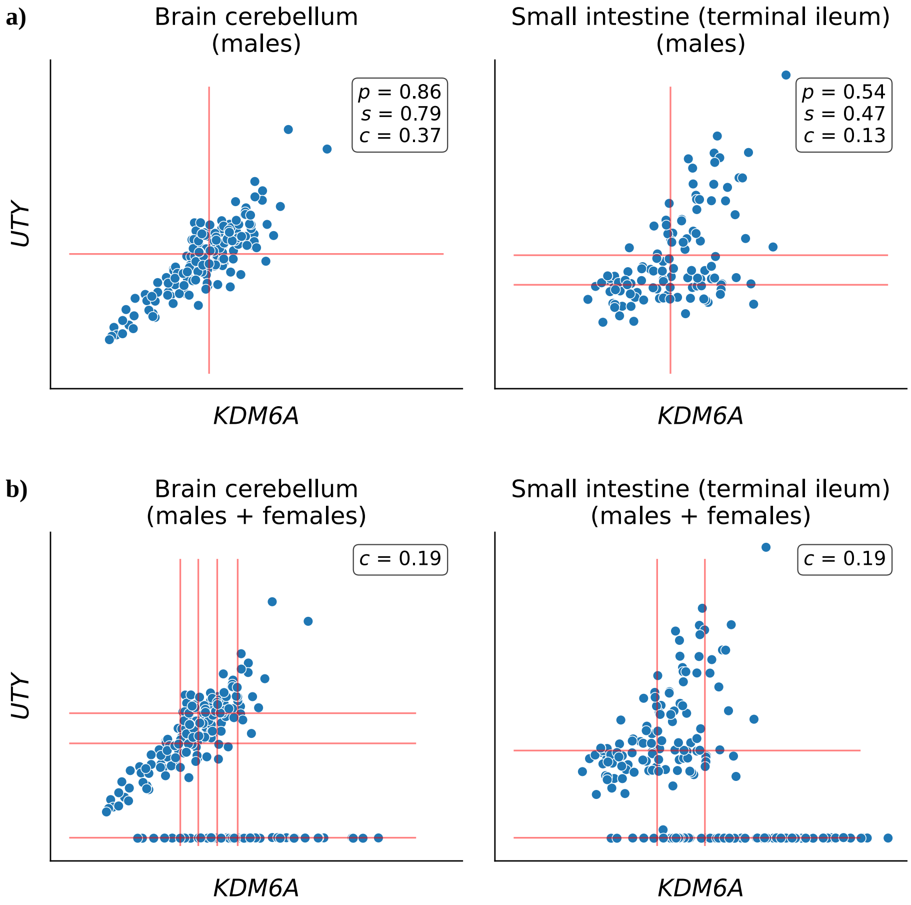

Figure S3: Linear and nonlinear patterns between *UTY* and *KDM6A* in brain cerebellum and small intestine (terminal ileum) in GTEx. a) Correlation values for Pearson, Spearman and CCC when only male samples are considered in brain cerebellum and small intestine. **b)** Correlation value of CCC when all samples (males and females) are considered. Vertical and horizontal red lines show how CCC clustered data points using each gene separately.

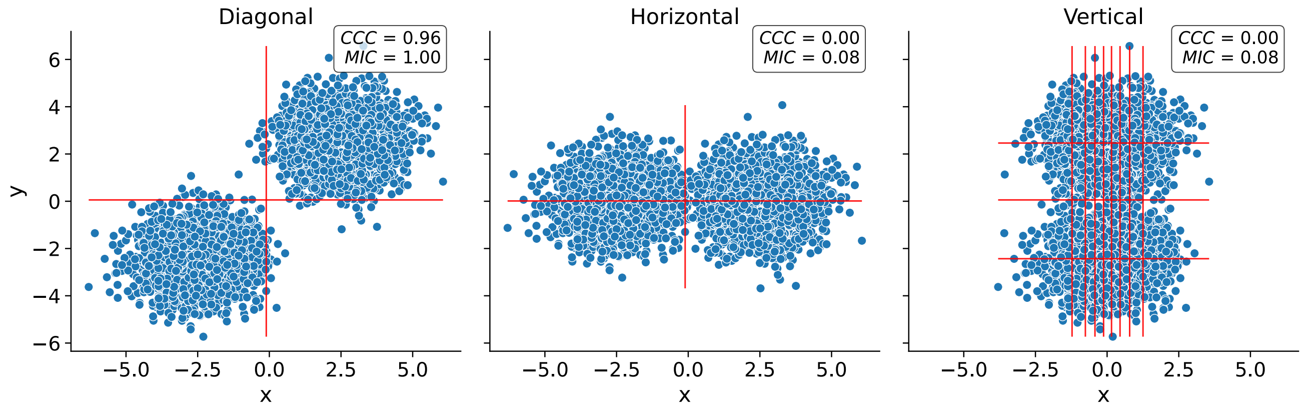

Figure S4: Behavior of CCC and MIC when substructure is present in the data. Two simulated, normally distributed clusters across two variables (x and y) are placed diagonally (left), horizontally (middle) and vertically (right), and the CCC and MIC values are calculated. Vertical and horizontal red lines show how CCC clustered data points using each variable separately.

Tables

Table S1: Network statistics of seven gene pairs shown in Figure 3b for blood and predicted cell types. Only gene pairs present in GIANT models are listed. For each gene in the pair (first column), the minimum, average and maximum interaction coefficients with the other genes in the network are shown.

	**Interaction confidence**						
	**Blood**			**Predicted cell type**			
**Gene**	**Min.**	**Avg.**	**Max.**	**Cell type**	**Min.**	**Avg.**	**Max.**
IFNG	0.19	0.42	0.54	Natural killer cell	0.74	0.90	0.99
SDS	0.18	0.29	0.41		0.65	0.81	0.94
PRSS36	0.07	0.10	0.14	Macrophage	0.04	0.05	0.08
CCL18	0.07	0.74	0.86		0.05	0.69	0.90
UTY	0.03	0.36	0.84	Placenta	0.01	0.03	0.04
KDM6A	0.03	0.42	0.58		0.04	0.38	0.61
DDX3Y	0.05	0.33	0.78	Testis	0.07	0.11	0.18
KDM6A	0.43	0.51	0.58		0.27	0.34	0.48
RASSF2	0.69	0.77	0.90	Leukocyte	0.66	0.74	0.88
CYTIP	0.74	0.85	0.91		0.76	0.84	0.91
MYOZ1	0.09	0.17	0.37	Skeletal muscle	0.11	0.11	0.12
TNNI2	0.10	0.22	0.44		0.10	0.11	0.12
SCGB3A1	0.16	0.19	0.23	Placenta	0.11	0.11	0.12
C19orf33	0.15	0.19	0.28		0.11	0.12	0.17
