# Supplementary material for: An efficient, not-only-linear correlation coefficient based on clustering
Source: Cell Syst. Author manuscript; Available in PMC 2025 Mar 28. (PMC11951854; doi:10.1016/j.cels.2024.08.005)
Supplement: MMC2 [file NIHMS2023070-supplement-MMC2.pdf]

Cell Systems Transparent Peer Review Record

An efficient not-only-linear correlation coefficient based on clustering

Milton Pividori, Marylyn D Ritchie, Diego H Milone, and Casey S Greene

Summary

Initial Submission:	Received Sept 9, 2022
	Preprint: doi.org/10.1101/2022.06.15.496326
	Scientific editor: Bernadett Gaal, DPhil
First round of review:	Number of reviewers: 2
	2 confidential, 0 signed
	Revision invited April 12, 2023
	Major changes anticipated
	Revision received June 18, 2024
Second round of review:	Number of reviewers: 1
	1 original, 0 new
	1 confidential, 0 signed
	Accepted Aug 15, 2024

This Transparent Peer Review Record is not systematically proofread, type-set, or edited. Special characters, formatting, and equations may fail to render properly. Standard procedural text within the editor's letters has been deleted for the sake of brevity, but all official correspondence specific to the manuscript has been preserved.

Editorial decision letter with reviewers' comments, first round of review

Dear Casey,

I hope this email finds you well. The reviews of your manuscript are back and I've appended them below. First of all, please accept once again my sincere apologies for the severe delay in the review process! As I previously mentioned, it was more of a challenge to recruit reviewers in this case than I had anticipated, and unfortunately we also had to recruit a new reviewer at a late stage. On balance, the reviewers appreciate the goals of the work presented here; they've provided constructive comments that are aligned with our hopes for the paper. Accordingly, we're happy to invite a revision that addresses the reviewers' concerns, particularly those of Reviewer 2.

I hope you find the reviewrs' feedback helpful. If you have any questions or concerns, I'm always happy to talk, either over email or Zoom. More technical information and advice about resubmission can be found below my signature. Please read it carefully, as it can save substantial time and effort later.

I look forward to seeing your revised manuscript.

All the best,
Bernadett

Bernadett Gaal, DPhil
Editor-in-Chief, Cell Systems

Reviewers' comments:

Reviewer #1: In "An efficient not-only-linear correlation coefficient based on machine learning: by Pividdori et al, the authors describe Clustermatch Correlation Coefficient (CCC) based on machine learning models. This algorithm is a useful advancement in detecting non-linear relationships in complex gene expression data which is critical for understanding complex information flow from the genome. I completely agree with this statement: "It is possible that the widespread use of linear coefficients may bias researchers away from genes with complex coexpression patterns."The software that implements this algorithm is available at https://github.com/greenelab/ccc under the BSD-2-Clause Plus Patent License. I have installed the software and it works as advertised.

The utility of the algorithm is proven by the results. For example, they found that "CCC behaved very similarly to MIC, although CCC was up to two orders of magnitude faster to run" indicating that it is supported by other approaches yet is computationally efficient. Further, the pairwise examples are compelling including the sex-dependent gene-gene relationships. I like the use of real (GTex) and simulated data.

Points for improvement:
1. The manuscript is well-written but could benefit from chunking up some huge paragraphs. For example, insert a paragraph break before "Here we introduce the Clustermatch Correlation Coefficient (CCC)".
2. It would be beneficial to the end-user to identify a significance threshold for the CCC metric on a large dataset? I think the authors use a ?80th percentile? A discussion of the rationale for thresholding would be ideal.
3. The algorithm details are complex and only discussed in the Methods. I would strongly suggest that the core concepts behind the algorithm be mentioned in the Abstract and be overviewed at the beginning of the Results.
4. (100% optional). Please check out an approach of similar spirit in this study: "Addressing noise in co-expression network construction" by Burns etal
https://academic.oup.com/bib/article/23/1/bbab495/6446269

Reviewer #2: The paper introduces the Clustermatch Correlation Coefficient (CCC), which is a new correlation coefficient based on clustering. The authors describe the method used to develop and test this coefficient, which involves clustering objects using each feature separately and computing the similarity between partitions using a measure such as the adjusted Rand index (ARI). The CCC is then computed as the maximum of similarity. (The GitHub repo is well organized, and I believe it's easy to reproduce this work). While the theoretical/statistical insight behind the method is not clearly articulated and seems intuition-based, the paper does provide some interesting results. However, in the absence of more formal analysis and results, it's hard to place why, how, and when CCC should be used. I summarize my major comments below:

Comments:
The statistical insight(s) behind the method is not very clear and seems to be largely intuition-based. It appears to offer an alternative way to calculate the MIC. It would be helpful to provide a theoretical perspective on the relationship between MIC and CCC. Clarify the differences between these two metrics.
The title of the paper does not accurately reflect the content, as it seems to focus more on clustering than machine learning. Consider revising the title to better represent the paper's focus.
The null distribution is important for any correlation metric, and without it, it's hard to interpret the results. Please provide information on how to compute the p-value for CCC.
Analyze the relationship between CCC and information-based metrics. Based on Figures 3 and 4, it seems that the main difference between CCC and Pearson/Spearman correlation is when the data has substructures, such as a mixture of two linear relationships. Is this correct?
In Figure 4, while there is a visible difference between the correlation of male samples, the CCC values are still quite close. For example, this can be observed in Brain Cerebellum and Small Intestine Terminal Ileum. Please address this.
Consider a scenario where there are two distinct clusters in the data. If the data has a clear cluster, the CCC will always be 1. This may not be an expected result, especially if we know there are substructures in the data, such as different cell types. Will the CCC method fail in such datasets?
In the algorithm pseudo-code, lines 2-5 are not easy to understand. It appears that line 4 is intended to get the partition. Please clarify how the partition is obtained and provide a more detailed explanation of the exact method used. If the partition is indeed an equal bin, the difference between MIC and CCC seems to be that CCC computes the ARI, while MIC computes the mutual information. Please confirm if this understanding is correct. If so, CCC may be an approximation of MIC.
Supplementary Note 2 mentions that Ref 75 is a faster method than MIC_e. What is the speed of the proposed method in comparison?

"Another advantage over MIC is that CCC can also process categorical variables together with numerical values." But how to get the cluster for categorical variables?

Authors' response to the reviewers' first round comments

Attached.

Editorial decision letter with reviewers' comments, second round of review

Dear Casey,

I'm very pleased to let you know that the reviews of your revised manuscript are back, the peer-review process is complete, and only a few minor, editorially-guided changes are needed to move forward towards publication. Unfortunately we did not hear back from Reviewer 2, but Reviewer 1 kindly additionally reviewed the revisions you have made in response to the other reviewer's concerns and we are happy to proceed.

I have made some editorial requests in the 'Editorial Notes' section below my signature. Please review it along with the Final Files Checklist and make the necessary changes. We've also put together this FAQ (click the Final Formatting Checks tab) for your convenience. Please ask any questions you may have, make any necessary changes to your manuscript files, and then upload your final files into Editorial Manager. Once we receive your formatted files, we will go through our formatting checks and let you know if further changes are needed.
We hope to receive your formatted files within 5 business days. Please email me directly if this timing is a problem or you're facing extenuating circumstances. Alternatively, if this manuscript needs to be officially accepted by a particular date because of grant deadlines, applications, or because it will help your trainees, please let me know.

Introducing new referencing style
To standardize the referencing style across Cell Press journals, starting from October 2022, we ask that all in-text citations be formatted as superscripted numbers (e.g. "Multiple reports support this observation.[1,2]"). Moving away from the Harvard referencing style (e.g. Smith *et al.*, 2020) will improve author and reader experiences. All manuscripts accepted from now on must use **the superscript numbered Cell Press referencing style**. Make sure to use this numbered referencing style for all new and revised submissions as well. Switching is easy. Just use the updated CSL and EndNote referencing styles for Cell Press articles.
Below my signature, you'll find specific information about what to expect next regarding formatting checks and working with our Production Department after acceptance. It's been a pleasure working with you, please feel free to contact our journal team with questions.

All the best,
Bernadett
Bernadett Gaál, DPhil
Editor-in-Chief, Cell Systems

Editorial Notes

Transparent Peer Review: Thank you for electing to make your manuscript's peer review process transparent. As part of our approach to Transparent Peer Review, we ask that you add the following sentence to the end of your abstract: "A record of this paper's Transparent Peer Review process is included in the Supplemental Information." Note that this ***doesn't*** count towards your 150 word total! Also, if you've deposited your work on a preprint server, that's great! Please drop me a quick email with your preprint's DOI and I'll make sure it's properly credited within your Transparent Peer Review record.

Manuscript Text:

- House style disallows editorializing within the text (e.g. strikingly, surprisingly, importantly, interestingly, etc.), especially the Results section. These terms are a distraction and they aren't needed—your excellent observations are certainly impactful enough to stand on their own. Please remove these words and others like them. "Notably" is suitably neutral to use once or twice if absolutely necessary.
- We don't allow "priority claims" (e.g. new, novel, etc.). For a discussion of why, read: http://crosstalk.cell.com/blog/getting-priorities-right-with-novelty-claims, http://crosstalk.cell.com/blog/novel-insights-into-priority-claims.
- Please double-check that you use the word "significantly" in the statistical sense only.

Figures and Legends:

Please look over your figures keeping the following in mind:

- Please ensure that all figures included in your point-by-point response to the reviewers' comments are present within the final version of the paper, either within the main text or within the Supplemental Information.

STAR Methods: Note that Cell Press has recently changed the way it approaches "availability" statements for the sake of ease and clarity. Please revise the first section of your STAR Methods as follows, noting that the particular examples used might not pertain to your study. Please consult the STAR Methods guidelines for additional information.

RESOURCE AVAILABILITY

Lead Contact: Further information and requests for resources and reagents should be directed to and will be fulfilled by the Lead Contact, Jane Doe (janedoe@qwerty.com).

Materials Availability: This study did not generate new materials. *-OR-* Plasmids generated in this study have been deposited at [Addgene, name and catalog number]. *-OR-* etc.

Data and Code Availability:

· **Source data statement** (described below)

· **Code statement** (described below)

· Any additional information required to reanalyze the data reported in this paper is available from the lead contact upon request.

Data and Code Availability statements **have three parts and each part must be present. Each part should be listed as a bullet point, as indicated above.**

Instructions for section 1: Data. The statements below may be used in any number or combination, but at least one must be present. They can be edited to suit your circumstance. Please ensure that all datatypes reported in your paper are represented in section 1. For more information, please consult this list of standardized datatypes and repositories recommended by Cell Press.

• This paper analyzes existing, publicly available data. These accession numbers for the datasets are listed in the key resources table.

Instructions for section 2: Code. The statements below may be used in any number or combination, but at least one must be present. They can be edited to suit your circumstance. ***If you are using GitHub, please follow the instructions here to archive a "version of record" of your GitHub repo at Zenodo, then report the resulting DOI. Additionally, please note that the Cell Systems strongly recommends that you also include an explicit reference to any scripts you may have used throughout your analysis or to generate your figures within section 2.***
• All original code has been deposited at [repository] and is publicly available as of the date of publication. DOIs are listed in the key resources table.
Instructions for section 3. Section 3 consists of the following statement: Any additional information required to reanalyze the data reported in this paper is available from the lead contact upon request.
In addition,
STAR Methods follows a standardized structure. Please reorganize your experimental procedures to include these specific headings in the following order: LEAD CONTACT AND MATERIALS AVAILABILITY (including the three statements detailed above); EXPERIMENTAL MODEL AND SUBJECT DETAILS (when appropriate); METHOD DETAILS (required); QUANTIFICATION AND STATISTICAL ANALYSIS (when appropriate); ADDITIONAL RESOURCES (when appropriate). We're happy to be flexible about how each section is organized and encourage useful subheadings, but the required sections need to be there, with their headings. They should also be in the order listed. Please see the STAR Methods guide for more information or contact me for help.
Please ensure that original code has been archived in a general purpose repository recommended by Cell Press and that its DOI is provided in the Software and Algorithms section of the Key Resources Table. If you've chosen to use GitHub, please follow the instructions here to archive a "version of record" of your GitHub repo at Zenodo, complete with a DOI. Thank you!
Cell Systems has taken the almost political stance that theory papers deserve the same "weight" as experimental papers, and we try very hard to treat both consistently. We believe that the deep mathematical exploration of problems that's usually found in a stand-alone Supplement is roughly equivalent to the Experimental Procedures section of experimental papers. Just as one must understand the methods used to appreciate the strengths and weaknesses of the data those methods generate, understanding the mathematics is necessary for understanding the Results in a deep way. For this reason, we ask that you move all of the current supplement into the Model Details section of the STAR Methods, and follow the rest of the STAR Methods instructions as well as you can.
Currently, you don't have a **Key Resources Table** (KRT). Note that the key resources table is required for manuscripts with an experimental component, and if a purely computational manuscript links to any external datasets (previously published or new), code-containing websites (e.g. a GitHub repo, noting that DOIs are strongly preferred), or uses non-standard software, it needs to include a key resources table that details these aspects of the paper. Purely computational or theoretical papers that don't contain any external links and use standard software don't require a key resources table, although you're welcome to include one if you like. For details, please refer to the Table Template or feel free to ask me for help.
Thank you!

Reviewer comments:

Reviewer #1: The authors have addressed my concerns.

Response to reviewers

Reviewers' comments are in black, and authors' responses start with "Authors' response" and use blue as the font color.

We include below a list of the major changes to our manuscript and method in light of reviewers' comments:

- We updated the description of our analyses and mentioned that although we always reported the statistical significance of the coefficients across all of our analyses, we focused on the strength of the association (i.e., the percentile of coefficient value).
- We updated Figure 1 (simulated data) and added the statistical significance for all variable pairs. We updated the caption and the text explaining this figure. The interpretation of the figure remains the same, with the added confidence of the statistical significance. In addition, Figure 1 was updated with simulated data incorporating categorical variables, which was suggested by a reviewer.
- We computed the p-value of CCC for a subset of gene pairs analyzed in the manuscript. We focused on gene pairs in each category in the "Disagreements" group of Figure 3a (right). These experimental details were added in the Methods section under "Statistical significance."
- We updated Figure 3b (GTEx, real dataset analyses) and now show the p-value for the selected gene pairs. We also replaced some gene pairs to more clearly show how using both the statistical significance and the strength of the correlation leads to the detection of meaningful patterns. For example, gene pairs such as *UTY* - *KDM6A* (included in the first submission) or *DDX3Y* - *KDM6A* (new in this resubmission) are prioritized by CCC (both high coefficient value and statistically significant) across all GTEx tissues (not only whole blood). However, as we note in the text, these gene pairs are not consistently detected by Pearson or Spearman across tissues.
- Our replication analyses (Figure 5) in GIANT now include the top gene pairs that are statistically significant only (FDR < 0.05). All the results and interpretations remain the same.
- We extended the Discussion section by adding other potential future improvements to make the computation of p-values faster (for instance, by limiting the number of runs via approaches based on extreme value theory).
- We added Supplementary Files with the coefficient values and p-values for the three correlation coefficients across all gene pairs analyzed.

Reviewer #1

In "An efficient not-only-linear correlation coefficient based on machine learning: by Pividdori et al, the authors describe Clustermatch Correlation Coefficient (CCC) based on machine learning models. This algorithm is a useful advancement in detecting non-linear relationships in complex gene expression data which is critical for understanding complex information flow from the genome. I completely agree with this statement: "It is possible that the widespread use of linear coefficients may bias researchers away from genes with complex coexpression patterns."The software that implements this algorithm is available at https://github.com/greenelab/ccc under the BSD-2-Clause Plus Patent License. I have installed the software and it works as advertised.

The utility of the algorithm is proven by the results. For example, they found that "CCC behaved very similarly to MIC, although CCC was up to two orders of magnitude faster to run" indicating that it is supported by other approaches yet is computationally efficient. Further, the pairwise examples are compelling including the sex-dependent gene-gene relationships. I like the use of real (GTex) and simulated data.

Authors' response: We thank the reviewer for the highly positive and constructive feedback. We put a lot of effort into making our software and pipelines reproducible, and we are glad that the reviewer could install and successfully test the package.

As part of this revision, we have significantly improved the performance of CCC, which is now approximately five times faster than the first version. This means that the difference with MIC is now larger regarding computational efficiency.

Points for improvement:

1. The manuscript is well-written but could benefit from chunking up some huge paragraphs. For example, insert a paragraph break before "Here we introduce the Clustermatch Correlation Coefficient (CCC)".

Authors' response: We thank the reviewer for these suggestions. As suggested, we split the paragraph mentioned in the Introduction by adding a break. We also found another large paragraph in the Results section ("We sought to systematically analyze…"), which we divided to improve clarity.

2. It would be beneficial to the end-user to identify a significance threshold for the CCC metric on a large dataset? I think the authors use a ?80th percentile? A discussion of the rationale for thresholding would be ideal.

Authors' response: We thank the reviewer for this important point we did not discuss in the first manuscript submission. In our analyses of real data with GTEx, we wanted to include the strongest relationships captured by the three coefficients. This is one common approach when building gene co-expression networks, where the largest coefficient values determine which gene pairs will be connected by an edge (via thresholding). Therefore, we selected the top 30% of gene pairs with the largest correlation values (i.e., values larger than the 70th percentile) for each coefficient. This gave us a trade-off between the strongest relationships and those in the limit between strongest and moderate.

However, we acknowledge that this selection is somewhat arbitrary. Therefore, we added the possibility to compute a p-value for CCC using permutation tests. Our package's "ccc" function now accepts a parameter "pvalue_n_perms" with the number of permutations to be computed. We now used the p-value across all our analyses.

We refer the reviewer to the list of changes at the top of this documen, which describes our manuscript updates to use permutation-computed p-values for gene pairs.

3. The algorithm details are complex and only discussed in the Methods. I would strongly suggest that the core concepts behind the algorithm be mentioned in the Abstract and be overviewed at the beginning of the Results.

Authors' response: We thank the reviewer for this feedback, which was very helpful in better communicating the key methodological aspects of CCC. We have improved the abstract by mentioning the core assumption of CCC, that *"if two features are similar, then the clustering of objects using each feature separately should match."* In the Results section, we have clarified some aspects of the method in the first subsection (which is now titled *"Overview of CCC: a not-only-linear correlation coefficient"*), and the Methods section now includes more statistical details about how the CCC is computed, important definitions and properties of CCC, better descriptions of the parameters and an updated description of the algorithm.

4. (100% optional). Please check out an approach of similar spirit in this study: "Addressing noise in co-expression network construction" by Burns etal https://academic.oup.com/bib/article/23/1/bbab495/6446269

Authors' response: We thank the reviewer for suggesting this interesting article. Although our study does not focus on constructing gene co-expression networks (GCN), our method CCC can be applied in this area. We completely agree with the article's statement that a *"'one-size-fits-all' approach to GCN construction is therefore problematic for large, multivariable datasets."* In our study, we used GTEx, which is a homogeneous dataset with expression measurements across different tissues but from the same individuals. In the future, users could apply CCC to more heterogeneous datasets, such as recount3, and combine it with the approaches mentioned in that article to build context-specific GCNs while addressing confounding variables. We have cited the article mentioned by the reviewer in our Discussion section.

Reviewer #2

The paper introduces the Clustermatch Correlation Coefficient (CCC), which is a new correlation coefficient based on clustering. The authors describe the method used to develop and test this coefficient, which involves clustering objects using each feature separately and computing the similarity between partitions using a measure such as the adjusted Rand index (ARI). The CCC is then computed as the maximum of similarity. (The GitHub repo is well organized, and I believe it's easy to reproduce this work). While the theoretical/statistical insight behind the method is not clearly articulated and seems intuition-based, the paper does provide some interesting results. However, in the absence of more formal analysis and results, it's hard to place why, how, and when CCC should be used. I summarize my major comments below:

Authors' response: We thank the reviewer for the positive and very constructive feedback, which was very helpful in improving our manuscript. We go into more depth below, but, in short, we have significantly improved the theoretical/statistical details about CCC (Methods section). We hope these new additions and updates will be useful to inform users about CCC.

Comments:
The statistical insight(s) behind the method is not very clear and seems to be largely intuition-based. It appears to offer an alternative way to calculate the MIC. It would be helpful to provide a theoretical perspective on the relationship between MIC and CCC. Clarify the differences between these two metrics.

Authors' response: We thank the reviewer for this important observation. We substantially expanded the subsection "The Clustermatch Correlation Coefficient (CCC)" in Methods with more details, which includes:

- the definitions of a partition, the adjusted Rand index (ARI), and the CCC,
- the properties of CCC derived from the ARI: symmetry, normalization, and constant baseline,
- an empirical analysis of how increasing values of the *k_max* parameter (the maximum number of clusters) affect CCC scores,
- details about the statistical significance, and
- an expanded section about our implementation of the algorithm.

Based on the extended details about CCC, we have also significantly expanded Supplementary Note 1: Comparison with the Maximal Information Coefficient (MIC). We comment on these changes in a related answer below.

The title of the paper does not accurately reflect the content, as it seems to focus more on clustering than machine learning. Consider revising the title to better represent the paper's focus.

Authors' response: We agree that the term machine learning in the title was too general, and therefore, we changed it to: "An efficient not-only-linear correlation coefficient based on clustering."

The null distribution is important for any correlation metric, and without it, it's hard to interpret the results. Please provide information on how to compute the p-value for CCC.

Authors' response: Deriving the distribution for CCC under the null is not straightforward since the method uses the maximum adjusted Rand Index across different pairs of partitions with different numbers of clusters. To address the question of p-values, we now use permutations to calculate them. Our Python package now accepts the parameter "pvalue_n_perms" and we have further improved the computational performance of CCC. It is now approximately five-fold faster than before, which helps when permutations are required.

We have updated the manuscript to use permutation-based p-values. We refer the reviewer to the list of changes at the top of this document, where we provide a list of changes describing our manuscript updates to use permutation-computed p-values for gene pairs.

Analyze the relationship between CCC and information-based metrics. Based on Figures 3 and 4, it seems that the main difference between CCC and Pearson/Spearman correlation is when the data has substructures, such as a mixture of two linear relationships. Is this correct?

Authors' response: As a not-only-linear correlation coefficient, CCC can capture patterns beyond linear ones, such as non-coexistence and quadratic relationships, and other complex patterns that embed two linear relationships with different slopes (Figure 1, bottom), as the reviewer mentions. We observed that CCC found some of these patterns in real data using GTEx whole blood. Therefore, the main difference between CCC and linear-only coefficients is that CCC can capture patterns beyond linear relationships. A *subset* of these nonlinear relationships is represented by different substructures or patterns in data, including a mixture of two linear relationships (such as gene pair *RASSF2* - *CYTIP* in Figure 3) or a mixture of a linear relationship and no relationship (such as gene pair *UTY* - *KDM6A*, with a linear pattern in males, and no pattern in females). Other nonlinear patterns could follow quadratic or other types of relationships where one variable is a function of the other, as we showed with simulated data (Figure 1, bottom). Therefore, CCC can capture complex relationships, including the cases of substructures in data. Our response regarding the relationship between CCC and information-based metrics such as MIC is included below.

In Figure 4, while there is a visible difference between the correlation of male samples, the CCC values are still quite close. For example, this can be observed in Brain Cerebellum and Small Intestine Terminal Ileum. Please address this.

Authors' response: This is an interesting point that helps clarify how CCC works. Figure 4 shows the relationships between *UTY* (chromosome Y) and *KDM6A* (chromosome X) across tissues in GTEx. For this gene pair, CCC can find a complex pattern where a subset of samples (males) follows a clear linear relationship, and there is no relationship in the rest of the samples (females). As the reviewer says, there is a difference in the strength of the linear correlation between male samples across different tissues. For example, in brain cerebellum, the linear correlation among male samples is stronger than in small intestine (terminal ileum). As shown in the figure below, this difference is reflected by all coefficients when only male samples are considered.

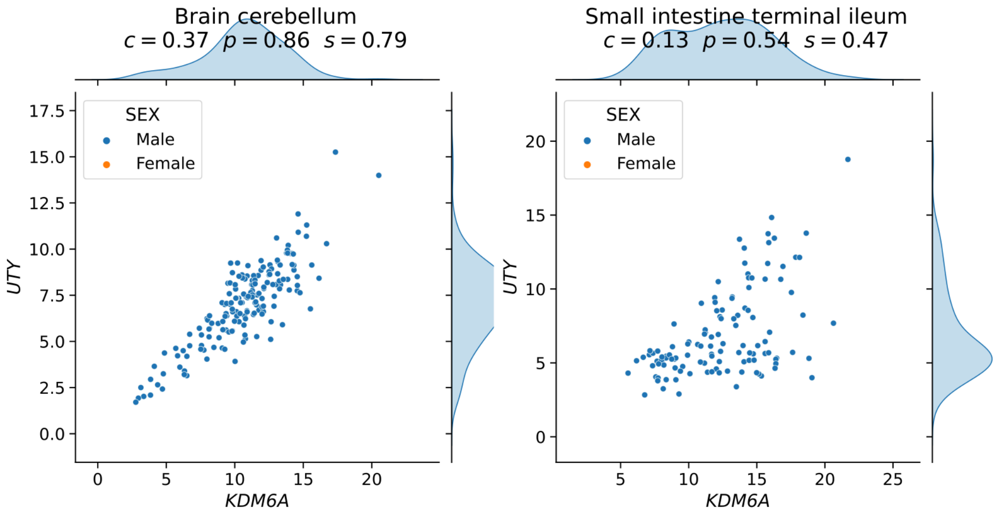

However, when we consider *all* samples (males and females), there is no longer a linear relationship between *UTY* and *KDM6A*. Therefore, while a subset of the data displays linear relationships, overall, it is no longer true that there is a linear correlation. CCC assumes that if two variables (genes in our case) are similar, the clustering of objects (samples) using each variable separately should match. This clustering of samples and their matching can be seen for the gene pair *UTY* / *KDM6A*: when we only consider male samples, CCC finds clusterings in brain cerebellum with a larger matching than in small intestine (terminal ileum) because the linear strength differs:

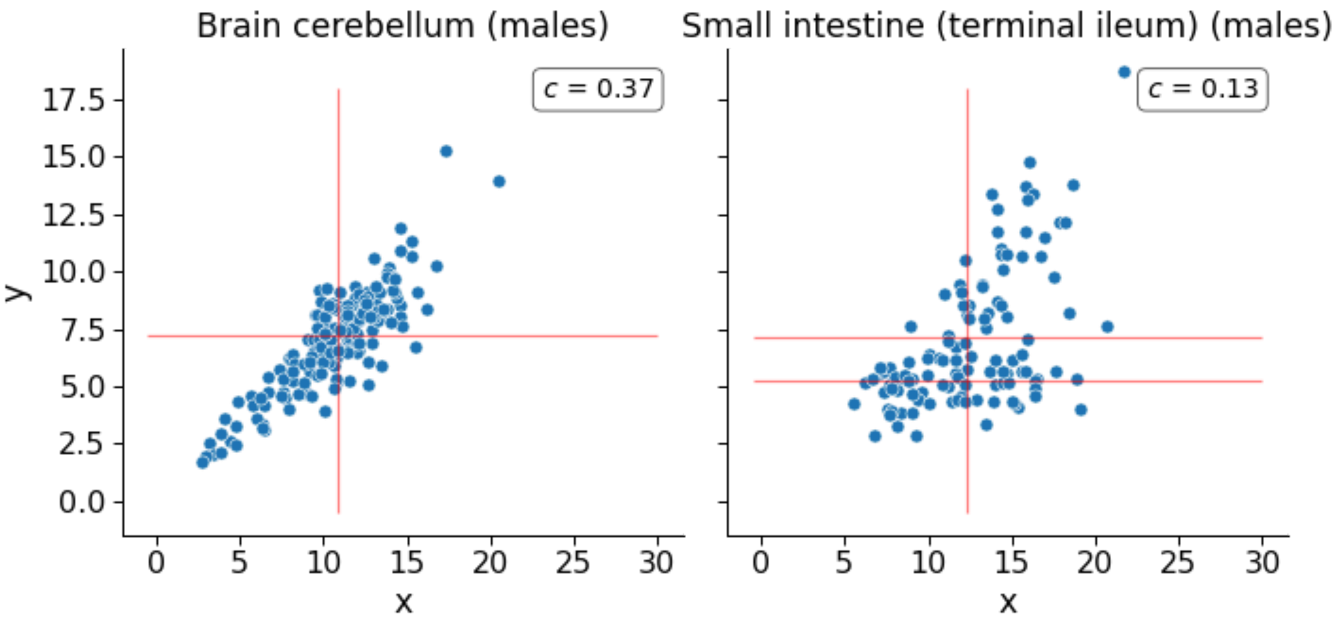

But when we consider *all* samples together (males and females), the pattern is nonlinear, the distribution of *all* the data is different, and so are the clusterings found by CCC:

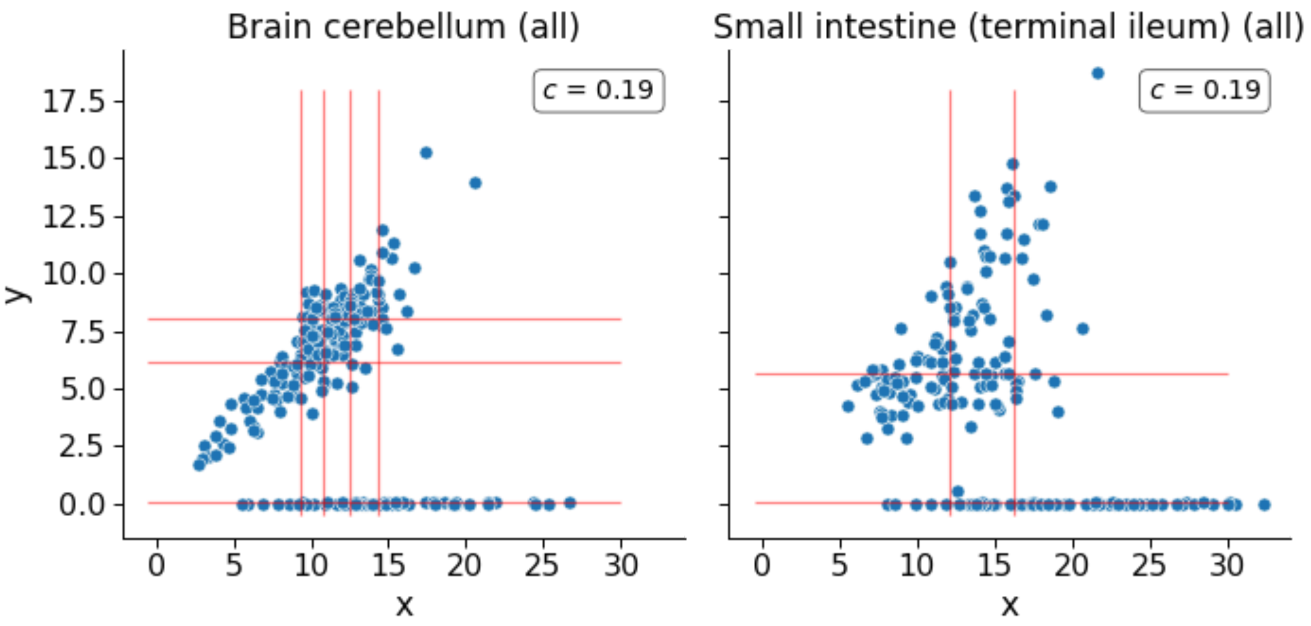

This effect of analyzing *all* the data (males and females) in this nonlinear pattern is also clear in the negative sign of Pearson and Spearman coefficients in small intestine or even other tissues with a very strong and clear linear pattern among male samples such as breast mammary tissue. In this case, this indicates that Pearson and Spearman, although statistically significant, are capturing the wrong pattern.

Therefore, the fact that CCC yields a similar value (0.19) for these two nonlinear patterns reflects a similar clustering matching when considering *all* the samples. When applied only to the data with linear relationships of varying strength, CCC performs consistently with other metrics.

Consider a scenario where there are two distinct clusters in the data. If the data has a clear cluster, the CCC will always be 1. This may not be an expected result, especially if we know there are substructures in the data, such as different cell types. Will the CCC method fail in such datasets?

Authors' response: CCC assumes that if two variables are similar, the clustering of objects using each variable *separately* should match. If the data has a clear structure with two clusters, the value of the CCC will depend on how those clusters are divided into groups using each variable separately and how those match. In the example below, we have simulated two distinct clusters (normally distributed), as the reviewer suggested, where clusters are placed diagonally, horizontally, and vertically.

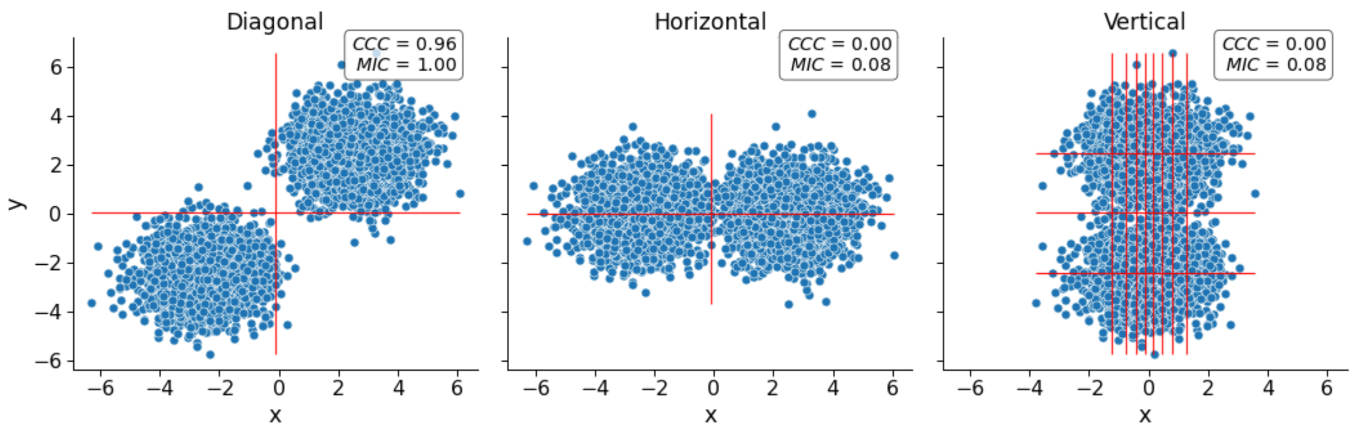

The only case where the CCC is close to 1.0 (Diagonal, left) is when the clusterings/partitions of objects using each variable (*x* and *y*) match, which coincides with a linear pattern. In the other two cases (Horizontal and Vertical), clusterings of objects do not match, leading to a CCC value of zero. We note that MIC has the same behavior.

Finally, the case of known and undesirable substructures in the data is an important point raised by the reviewer. We now note this aspect in the discussion as part of the limitations of our analyses.

In the algorithm pseudo-code, lines 2-5 are not easy to understand. It appears that line 4 is intended to get the partition. Please clarify how the partition is obtained and provide a more detailed explanation of the exact method used. If the partition is indeed an equal bin, the difference between MIC and CCC seems to be that CCC computes the ARI, while MIC computes the mutual information. Please confirm if this understanding is correct. If so, CCC may be an approximation of MIC.

Authors' response: This is an important question that helps further clarify how CCC works. We have significantly improved the Methods section with more details about CCC, including definitions and its properties, and a better description of the algorithm and how partitions are derived. This allowed us to better compare with MIC.

Regarding how a partition is obtained, in Algorithm 1, lines 2-6 in function *get_partitions* derive different partitions from a numerical variable across a range of *k* values (number of clusters). Line 4 gets the quantiles for a specific *k*, while line 5 uses these quantiles to derive a partition. For example, when *k*=2, the median is used (line 4) to separate objects (line 5); when k=3, the 33th and 66th percentiles are used to separate objects, and so on. The description of this procedure and many other details about CCC have been significantly improved in the Methods section, under the subsection *"The Clustermatch Correlation Coefficient (CCC)."* We included a subsection titled *"Definitions,"* defining a partition of *n* objects from a numerical and categorical variable.

Regarding the similarities and differences between CCC and MIC, these two coefficients are similar in several aspects: CCC and MIC are symmetric and normalized between zero and one; both perform some kind of binning to the input vectors; and both have a similar parameter that controls the complexity of the patterns found and computation time. However, despite these shared characteristics, we believe they are fundamentally different across several aspects:

- <u>CCC, grounded in clustering approaches, is conceptually different</u>. The clustering process in CCC effectively transforms each variable into a set of partitions containing a different number of clusters. The CCC then quantifies the correlation between variables by assessing the similarity of these partitions. This allows the processing of different types of variables, including *both numerical and categorical variables*, even when the categories are nominal (i.e., they lack intrinsic order), as explained in Methods. MIC, however, is specifically designed for numerical variables.
- <u>The CCC's approach to partitioning the data is more general than MIC's</u>. The reviewer asks how a partition is obtained in CCC and how this compares with MIC. Both CCC and MIC involve binning the input data vectors, aiming to maximize the mutual information and the ARI, respectively. However, the partitioning approaches in both methods are similar only when considering 1-dimensional variables (such as correlating gene pairs). In theory, the clustering-based approach in CCC should also support correlating variables with arbitrary dimensions. If *x* and *y* have 2 and 4 dimensions, respectively, CCC only needs partitions from them, which could be obtained by applying a standard clustering algorithm

such as k-means. This is enabled by the conceptually different and more general partitioning approach of CCC.

- CCC and MIC optimize different measures with very different properties. MIC uses a normalized mutual information score, whereas CCC uses the adjusted Rand index. The differences between these two measures are important. Although both are symmetric and normalized, the ARI has an advantageous property: it consistently returns a baseline (zero) for independently drawn partitions, irrespective of the number of clusters (see our new Figure S4). This property is not inherent in mutual information, which can produce varied values for independent variables if the grid dimensions vary. To mitigate this, MIC needs to limit the grid size.
- The maximum number of partitions does not constrain CCC to capture more complex patterns. Regarding their parameters, CCC's *k_max* (maximum number of clusters) and MIC's *B(n)* (maximum grid size) serve similar purposes. They control both the complexity of the patterns detected and the computational time. However, a critical distinction exists between the two: the constant baseline property of ARIs ensures that CCC returns a value close to zero for independent variables, regardless of *k_max*. In contrast, MIC may produce non-zero scores for independent data if *B(n)* is set too high, as discussed in Section 2.2.1 of the supplementary material in Ref 24.

Therefore, although CCC and MIC share some characteristics, we believe they are fundamentally different across different aspects. Thanks to the suggested changes by the reviewer, our manuscript now includes more details about CCC, which allowed us to better conceptually compare it with MIC. In addition to extending the Methods section about CCC, we have added a new subsection in the Supplementary Material titled *"Conceptual and statistical differences between CCC and MIC."*

Supplementary Note 2 mentions that Ref 75 is a faster method than MIC_e. What is the speed of the proposed method in comparison?

Authors' response: In Supplementary Note 2, we say that the use of MIC on large datasets *"remains limited due to its very long computation time,"* and then we cite some methodological/implementation improvements, including Ref 75 (which proposes the BackMIC algorithm to estimate the MIC; in the revised paper this is now Ref 76) mentioned by the reviewer. We did not intend to convey that BackMIC is faster than MIC_e; BackMIC is barely faster than the *original* MIC implementation (not MIC_e) on independent data and slower with a variable pair containing a parabola (see Table 3 in that paper). All the other articles we cite in Supplementary Note 2 are prior to the publication of MIC_e (Ref 73, published in 2016). To our knowledge, MIC_e represents the latest version of the original MIC, providing improved consistency and runtime complexity over the original implementation (Ref 37, published in 2011). For this reason, we decided to compare CCC with MIC_e, which is the latest and improved implementation of MIC, and it was developed by the original authors.

One potential source of confusion is that, in our original submission, we cited the MIC_e article as published in 2010 instead of 2016. We apologize for this error that confused the timing of the different MIC-based implementations in the literature.

"Another advantage over MIC is that CCC can also process categorical variables together with numerical values." But how to get the cluster for categorical variables?

Authors' response: We thank the reviewer for this question. Internally, CCC converts all variables (numerical or categorical) into a set of partitions of the data objects. For numerical variables, we use different quantiles (e.g., the median separates numerical data into two clusters). For categorical variables, we use the category values themselves as the natural cluster indicators (e.g., if the feature "color" has three values, "red," "green," and "blue," then the data will be clustered into those three colors). Finally, when we have all the partitions for

each variable, regardless of their data type, we compare them using the adjusted Rand Index to derive a similarity between them. This is how CCC can process numerical and categorical variables together without requiring any transformation or mapping process.

However, although we claimed that CCC can process both numerical and categorical data together, we did not test this in our original submission. For this resubmission, we extended our simulated examples in the first section of Results (*"Overview of CCC: a not-only-linear correlation coefficient"*) to include categorical variables. For this, we added a new panel (Figure 1c) with a mix of numerical and categorical variables and updated the text. We also extended the Methods section to define how categorical variables are partitioned and provide more details about the algorithm.
